# Transcriptomic Analysis Identified ARHGAP Family as a Novel Biomarker Associated With Tumor-Promoting Immune Infiltration and Nanomechanical Characteristics in Bladder Cancer

**DOI:** 10.3389/fcell.2021.657219

**Published:** 2021-07-07

**Authors:** Chen Yang, Siqi Wu, Zezhong Mou, Quan Zhou, Zheyu Zhang, Yiling Chen, Yuxi Ou, Xinan Chen, Xiyu Dai, Chenyang Xu, Na Liu, Haowen Jiang

**Affiliations:** ^1^Department of Urology, Huashan Hospital, Fudan University, Shanghai, China; ^2^Fudan Institute of Urology, Huashan Hospital, Fudan University, Shanghai, China; ^3^National Clinical Research Center for Aging and Medicine, Fudan University, Shanghai, China; ^4^School of Mechatronics Engineering and Automation, Shanghai University, Shanghai, China

**Keywords:** bladder cancer, ARHGAP, immune infiltration, tumor microenvironment, cellular mechanical properties

## Abstract

Bladder cancer (BCa) is a common lethal urinary malignancy worldwide. The role of ARHGAP family genes in BCa and its association with immuno-microenvironment remain largely unknown. ARHGAP family expression and immune infiltration in BCa were analyzed by bioinformatics analysis. Then, we investigated cell proliferation, invasion, and migration *in vivo* and *in vitro* of the ARHGAP family. Furthermore, atomic force microscopy (AFM) was employed in measuring cellular mechanical properties of BCa cells. The results demonstrated that ARHGAP family genes correlate with a tumor-promoting microenvironment with a lower Th1/Th2 cell ratio, higher DC cell infiltration, higher Treg cell infiltration, and T-cell exhaustion phenotype. Silencing ARHGAP5, ARHGAP17, and ARHGAP24 suppressed BCa cell proliferation, migration, and metastasis. Knocking down of ARHGAPs in T24 cells caused a relatively higher Young’s modulus and lower adhesive force and cell height. Taken together, ARHGAP family genes promote BCa progressing through establishing a tumor-promoting microenvironment and promoting cancer progression.

## Introduction

Bladder cancer (BCa) is the ninth leading diagnosed malignancy, which causes 17,980 mortality worldwide in 2020 (Siegel et al., 2020). The majority of BCa cases are classified as non-muscle invasive BCa representing the curable pathological type, while the other 25% are muscle-invasive BCa characterized by rapid progression and high recurrence rate, and eventually progress into metastatic disease (Smith et al., 2012; Nekolla et al., 2019). In recent years, various attempts to detect BCa at an early stage or explore the potential mechanism of lethal BCa have been made (Chen et al., 2020; Xie et al., 2021), so treatment strategies based on underlying molecular mechanisms in the metastasis and progression of BCa is in urgent need (Duggan et al., 2000).

Cancer cells are endowed with unique biological capabilities allowing them for constitutive survival, proliferation, and invasion ability during carcinogenesis (Chaffer and Weinberg, 2011). Adaptive immune response, a key regulator in regulating oncogenesis, has been well studied in the past decades. Several cancer-related hallmarks have been found responsible for the interaction of tumor and immune cells. Some of them can impair the differentiation and maturation of various immune cell subpopulations, which eventually leads to weakened antitumor functions in the tumor microenvironment, helping cancer cells to escape from immune surveillance (Chen and Mellman, 2017). In several tumor types, a better prognosis is mainly associated with infiltration of CD8+ T cells, type 1 T helper (Th1) cells, natural killer (NK) cells, and M1 macrophages. Conversely, poor prognosis usually correlates with high infiltration of Treg cells, Th2 cells, MDSCs, and M2 macrophages, and neutrophils (Fridman et al., 2012; Becht et al., 2016). These findings implied a distinctive mechanism of BCa-related immunological regulation, which could bring a radical revolution in BCa treatment other than the classical cisplatin-based chemotherapy.

The Rho GTPase family, a part of the Ras superfamily, consists of some highly conserved genes in regulating biological processes like cytoskeleton organization, vesicle trafficking, cell cycle, cell polarity, cell invasion, and cell migration (Haraguchi et al., 2019). Rho GTPases catalyze the conversion between active form and inactive form of Ras superfamily, henceforth, suppressing Rho GTPase downstream cellular biological processes. GTPase-activating proteins (RhoGAPs), which negatively regulates Rho GTPases are known as ARHGAP family (Post et al., 2013). While the roles of several ARHGAP family members have been identified in several types of cancer (Gen et al., 2009; Wang et al., 2014a, b; Luo et al., 2016; Hashimoto et al., 2018), the function of ARHGAP family in BCa, especially in immunological microenvironment of BCa, has not been elucidated.

Through comprehensive assessment of ARHGAP genes, we found a lower ARHGAP family expression in BCa, of which ARHGAP5, ARHGAP8, ARHGAP11A, ARHGAP17, ARHGAP24, ARHGAP37 (STARD13), and ARHGAP38 (STARD8) were highly related with prognosis. Axon guidance, focal adhesion, and leukocyte transendothelial migration were associated with the ARHGAP family genes, while focal adhesion, chemokine signaling pathway, and T-cell receptor signaling pathway were enriched in ARHGAP coexpression genes. We demonstrated that the ARHGAP family genes correlate with a tumor-promoting microenvironment. Silencing ARHGAP5, ARHGAP17, and ARHGAP24 suppressed BC cell proliferation, migration, and metastasis. Meanwhile, we validated that knocking down of ARHGAPs in T24 cells caused a relatively higher Young’s modulus and lower adhesive force and cell height, suggesting that ARHGAP could maintain a malignant cellular mechanical property of BCa. Our work offers novel insights into a potential mechanism of ARHGAP family genes’ correlation with immune microenvironment in BCa.

## Materials and Methods

### cBioPortal Database Analysis

The cBioPortal database^1^ offers visualized analysis of cancer genomics based on available high-throughput sequencing data (Cerami et al., 2012). To analyze the mutation of the ARHGAP family genes, a network of the mutation profile of ARHGAP family was generated based on data of 413 samples in The Cancer Genome Atlas database (TCGA, Bladder Cancer, Cell, 2017) (Robertson et al., 2017) in cBioPortal.

### Oncomine Database Analysis

The Oncomine database (Rhodes et al., 2004)^2^ compiled 86,733 cancer samples within 715 gene expression into construction of a comprehensive data-mining database. We assessed the ARHGAP family expression in various kinds of tumor types.

### TIMER Database Analysis

The TIMER^3^ database contributes to analyzing immune cell infiltration level in multiple cancers through pathological validation and improved statistical methodology to evaluate tumor immune infiltration (Li et al., 2017). We employed this database to assess differences in expression level of several candidates in the ARHGAP family in BCa to explore the association between ARHGAP family expression and infiltration level of particular immune cell subsets including B cells, CD4+ T and CD8+ T cells, neutrophils, macrophages, and dendritic cells. The Kaplan–Meier curve is also applied to analyze patient survival of the differential expression of the ARHGAP family gene-associated immune cell infiltration. The correlation between candidate ARHGAP family genes expression and gene markers of immune cells through relevant modules were analyzed.

### GEPIA Database Analysis

The GEPIA database (Tang et al., 2017)^4^ facilitates the standardized analysis of RNA-seq data from 9,736 tumors and 8,587 normal samples originating from The Cancer Genome Atlas (TCGA) and the Genotype-Tissue Expression (GTEx) data sets. We therefore assessed the link between the ARHGAP family gene expression and patients’ prognoses in BCa.

### Gene Set Enrichment Analysis

To further investigate the biological concepts associated with the ARHGAP family genes, we performed Gene Set Enrichment Analysis (GSEA) on the RNA-seq data downloaded from TCGA database. The mRNA expression of BCa was divided into high and low groups according to expression level. The carcinoma-related pathways gene sets (h.all.v6.0.symbols.gmt) were downloaded from the Molecular Signatures Database-MsigDB^5^. Enrichment analysis was performed by randomly repeating 1,000 times. Gene sets with a *p*-value < 0.05, normalized enrichment score (NES) > 1 or < -1, and false discovery rate (FDR) < 0.25 were considered as significantly enriched.

### Correlation and Protein Interaction Analysis

We both performed correlation and protein–protein interaction (PPI) analysis on the ARHGAP family genes. Herein, we generated correlation analysis by using BCa’s mRNA expression of the ARHGAP family genes downloaded from TCGA database. The results of correlation and relative *p*-value were constructed by using “corrplot” package in R (version 4.0.2). Then we applied GeneMANIA^6^ to analyze the ARHGAP family gene interaction and showed the PPI network including coexpression, pathway, predicted, colocalization, and genetic interactions. We also explored 50 most potentially relevant proteins by STRING^7^ database to serve the PPI network and showed the MCODE components identified by Metascape^8^.

### Gene Ontology and Kyoto Encyclopedia of Genes and Genomes Functional Enrichment Analysis

To explore the biological functions and novel pathways of the ARHGAP family genes, we applied Gene Ontology (GO) enrichment and Kyoto Encyclopedia of Genes and Genomes database (KEGG) enrichment. All GO and KEGG functional enrichment analysis were carried out by “clusterProfiler” and “enrichplot” package in R (version 4.0.2). Results of GO enrichment analysis including “Biological Process,” “Cellular Component,” and “Molecular Function” were visualized as a dot plot. Several most representative GO and KEGG analysis were also shown by R (version 4.0.2). A *p*–value < 0.05 was set as the cutoff criterion and considered as statistically significant.

### Tumor-Infiltrating Immune Cell Portraying

Two methods, xCell (Aran et al., 2017) and TIMER (Tumor Immune Estimation Resource,^9^ were used to analyze the tissue-infiltrating cell-type abundance from bulk RNA-seq data. Pre-calculated TCGA data by xCell was downloaded from the xCell web tool^10^. Cell type enrichment scores of TCGA-BLCA samples were extracted as reflection of certain cell type abundance. Metrics including immune score, stroma score, and microenvironment score were defined according to the source code of xCell on GitHub. Among 64 cell types xCell could output, cell types including CD8+ T cells, NK cells, dendritic cells (DCs), CD4+ T cells, regulatory T cells (Tregs), macrophage M1, macrophage M2, type 2 T-helper cells (Th2 cells), and type 1 T-helper cells (Th1 cells) were chosen to perform further analysis. R package “ggpubr” was used to visualize enrichment scores between groups. Samples with certain gene expression level (FPKM normalized expression level) higher than the median of that of all selected samples were defined as highly expressed, while the remaining samples as lowly expressed. Wilcoxon signed rank test was used to compare means between two groups. “Gene module” of TIMER was used to explore the correlation between gene expression and abundance of immune infiltrates, with BLCA (bladder urothelial carcinoma) selected as cancer types.

### Patient Samples

The study protocol was approved by the Ethics Committee of Huashan Hospital (Shanghai, China; approval no. KY2011-009) and conducted in accordance with the tenets of the Declaration of Helsinki. All patients consented to the use of resected tissues for research purposes.

A total of 90 pairs of BC tissues and adjacent tissues were collected for tissue microarray (TMA) construction from BC patients after surgical treatment in Huashan Hospital, Fudan University, between January 2007 and January 2013 including a 5-year follow-up. Two experienced pathologists confirm the pathological diagnoses of BC according to the 7th edition of the TNM classification of the Union for International Cancer Control (Wittekind et al., 2019).

Another seven pairs of high-grade and seven pairs of low-grade bladder cancer with normal samples were obtained from Huashan Urology Tissue Bank under an approval from the Ethics Committee of Huashan Hospital. Samples were harvested and immediately snap frozen in liquid nitrogen. Meanwhile, 20 fresh bladder cancer tumor tissues were obtained for flow cytometry.

### Immunohistochemical Staining

Single immunohistochemical staining was performed on TMA slides and 20 FFPE tissues from fresh resected tumors. The immunohistochemical staining procedure is illustrated as below. The slides were deparaffinized and rehydrated in advance with dimethylbenzene and ethanol. Heated sodium citrate buffer (0.01 M, pH = 6) was then applied for the slides for antigen repair. The slides were incubated in normal equine serum for 1 h at 37°C for blocking non-specific binding. The slides were washed and incubated with CD8 (Abcam, ab17147), CD4 (Abcam, ab67001), FOXP3 (Abcam, ab22510), ARHGAP5 (Abcam, ab32328), ARHGAP17 (Abcam, ab229221), and ARHGAP24 (Abcam, ab203874) overnight at 4°C in a wet chamber. The slides were stained with horseradish peroxidase-conjugated secondary antibody for 1 h at room temperature and then developed with DAB and hematoxylin. For the quantification of immunohistochemical staining, three randomized high-power fields of each sample were quantitated for the positive-staining cells, and the mean value was adopted. We quantitated FOXP3+ cells in each whole section since its relatively low infiltration in tumor. The 90 samples of the TMA slides were evenly divided into the ARHGAP family high/low groups according to the median value of ARHGAP5+, ARHGAP17+, and ARHGAP24+ cells. Then the three cutoff values were applied for the 20 fresh tumor tissues to define the ARHGAP family high/low groups.

### Cell Culture and Infection

Bladder transitional cell carcinoma cell lines (RT4, UM-UC-3, T24, 5,637, and J82), immortalized uroepithelium cell line (SV-HUC-1), and human embryonic kidney cells (HEK-293) were received from Shanghai Yuanye Bio-Technology Co. (Shanghai, China). All cells were maintained at 37°C with 5% CO_2_ in Dulbecco’s modified Eagle’s medium (DMEM; Gibco, New York, NY, United States) supplemented with 10% fetal bovine serum (FBS; Gibco, New York, NY, United States). To knock down ARHGAP5, 17, 24, the recombinant plasmid vector or control was purchased from Vigene (Jinan, China). HEK-293T cells were seeded into a 10-cm culture dish 24 h before transfection with PSPAX2 and PMD2G. The medium containing virus particles was collected at 72 h post-transfection. After filtration (0.45-μm filter), the medium was added to T24 and UM-UC-3 for viral infection with fresh medium replenished 24 h later. After 48 h postinfection, selection was done with 1.0 μg/ml of puromycin (Sangon, China).

### Cell Proliferation Assay

For colony formation assay, cells were seeded into six-well plates at a density of 600 cells per well. After 7–9 days of incubation, cells were fixed with 4% (w/v) paraformaldehyde (PFA) and stained with crystal violet solution.

### 5-Ethynyl-2′-Deoxyuridine Assay

5-Ethynyl-2′-deoxyuridine (EdU) assay kit (RiboBio, Guangzhou, China) was used to quantitatively investigate cell proliferation through DNA synthesis. A Nikon microscope (Nikon, Tokyo, Japan) was used to measure DNA synthesis reflected by red signals.

### Atomic Force Microscopy

The mechanical properties of cells were quantified using a commercial atomic force microscopy (AFM) instrument (BioScope Resolve; Bruker Corporation, Billerica, MA, United States). For characterization, dishes containing cultured primary cells were placed on a stage equipped with a vacuum pump with low noise and drum effects. A PeakForce Quantitative Nanomechanical Mapping—Live Cell probe (Bruker Corporation), with a tip length of 17 μm, tip radius of 65 nm, tip half angle of 18°, and spring constant of 0.076 N/m, was applied to probe the cell surface of the contact model. For characterization, a constant loading force of 1 nN was applied. Deflection images (32 × 32 pixels) were acquired at a ramp rate of 10 Hz and a ramp area of 5 μm × 5 μm. Automatic gain control was used to improve the feedback for surface tracking. YM and AF signal channels were used to map the cell force. All experiments were completed within 2 h to ensure cellular health, and the nanomechanical properties were determined from the AFM force map using Nanoscope Analysis software v1.80 (Bruker Nano Surfaces, Goleta, CA, United States).

### Transwell Assay

Cell migration was analyzed using Transwell chambers (Corning, New York, NY, United States) in accordance with the manufacturer’s protocol. After incubation for 24 h, the cells on the upper surfaces of the Transwell chambers were removed, and the cells located on the lower surfaces were fixed with 4% PFA, followed with crystal violet staining. The stained cells were photographed and counted in five randomly selected fields.

### Western Blot Analysis

Lysates from cells and tumor tissues were prepared to determine protein levels using the Bradford assay (Bio-Rad). Proteins were separated by 10% SDS-PAGE and transferred to polyvinylidene difluoride transfer membranes. The blots were blocked with freshly prepared 5% non-fat milk in PBST for 2 h at room temperature. Then the blots were incubated at 4°C overnight with primary antibodies. After washing with PBST, the blots were incubated with horseradish peroxidase-conjugated (HRP-conjugated) donkey anti-rabbit IgG or sheep anti-mouse IgG (Invitrogen, Shanghai, China) at room temperature for 2 h. ECL substrate (CLiNX, Shanghai, China) was used for detecting HRP-conjugated antibody.

### Xenograft Mouse Model and Metastasis Model

Male BALB/c nude mice (5 weeks old) were obtained from SLACOM (Shanghai, China) and used as xenograft hosts. Nude mice were maintained under a specific pathogen-free condition with approval of the Animal Care Committee of Fudan University.

For subcutaneous tumor formation assay, 1 × 107 transfected T24 cells were suspended in 0.2 ml of PBS and subcutaneously injected (*n* = 6 for each group). From day 4 on, tumor size was measured every 5 days by a caliper and was recorded as volume (1/6 × Π × L × W × H). The mice were sacrificed for dissection 21 days after injection.

For metastasis analyses, 1 × 105 transfected T24-luc cells were intravenously injected into mice tails (*n* = 6 mice per group). After 28 days, mice were intraperitoneally injected with 150 mg D-luciferin/kg body weight (Beyotime, Shanghai, China), then tumor development was monitored by bioluminescence imaging with the IVIS Spectrum *in vivo* Imaging System and Living Image software (PerkinElmer, Waltham, MA, United States).

### Flow Cytometry

Twenty fresh resected bladder cancer tissues were obtained from Huashan Hospital. The flow cytometry procedure is illustrated below. Tumor tissues were digested with 1 mg/ml of collagenase V (Sigma, St. Louis, MO, United States, C9263) and 0.2 mg/ml of DNase I (Sangon Biotech, Shanghai, China, A610099) for 12 h, incubated in RBC lysis buffer for 5 min and then filtered through a 70-μm strainer to collect single-cell populations. The single cell suspensions were stained with membrane antibodies including CD45 (BD Biosciences, Franklin Lakes, NJ, United States, 557833), CD3 (BD Biosciences, 552852), CD8 (BD Biosciences, 564526), PD-1 (BD Biosciences, 561272), Tim-3 (BD Bioscience, 565558), and LAG-3 (BD Biosciences, 565616) for 30 min at 4°C after Fc receptor blocking. The single-cell suspensions were then treated with BD Fixation/Permeabilization Solution Kit with BD Golgistop (BD Biosciences, 554715) to stain intracellular proteins including IFN-γ (BD Biosciences, 557643), GZMB (BD Biosciences, 560212), and perforin (BD Biosciences, 556437). Cells were collected and then analyzed on a BD FACSVerse Flow Cytometer (BD Biosciences, Franklin Lakes, NJ, United States).

### Statistical Analysis

The databases in our research were used for generating survival plots to analyze HR and *p*-value through log-rank test. Spearman’s correlation was used to gauge correlation between particular variables.

## Results

### Dysregulated Mutation and Expression of Rho-GTPase-Activating Proteins Genes Correlate With RFS and OS in Bladder Cancer Patients

**FIGURE 1:**
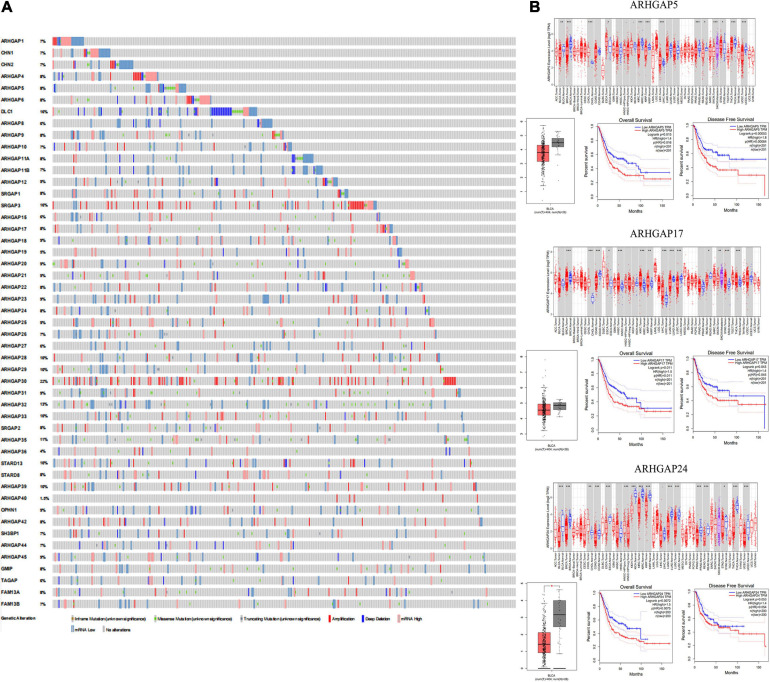
Identification of expression profile and prognosis related to the Rho-GTPase-activating proteins (ARHGAP) family gene in bladder cancer (BCa). **(A)** ARHGAP mutation level in BCa according to the analysis of cBioPortal. **(B)** Expression level of ARHGAP5, ARHGAP17, and ARHGAP24 in several tumor and normal tissues and Expression, OS and DFS of ARHGAP5 in BCa.

With the discovery and validation of Rho GTPase-activating proteins, we merely included ARHGAP1 to ARHGAP49 in our work with the summary of subfamily in the ARHGAP family genes (Table 1 and Figure 1A).

**TABLE 1:** Summary of sub-family in ARHGAP family genes.

	Subfamily	Description	Alias
ARHGAP1		Rho GTPase activating protein 1	CDC42GAP, RHOGAP, RHOGAP1, p50rhoGAP
ARHGAP2	CHN1	chimerin 1	ARHGAP2, CHN, DURS2, NC, RHOGAP2
ARHGAP3	CHN2	chimerin 2	ARHGAP3, BCH, CHN2-3, RHOGAP3
ARHGAP4		Rho GTPase activating protein 4	C1, RGC1, RhoGAP4, SrGAP4, p115
ARHGAP5		Rho GTPase activating protein 5	GFI2, RhoGAP5, p190-B, p190BRhoGAP
ARHGAP6		Rho GTPase activating protein 6	RHOGAP6, RHOGAPX-1
ARHGAP7	DLC1	DLC1 Rho GTPase activating protein	ARHGAP7, HP, STARD12, p122-RhoGAP
ARHGAP8		Rho GTPase activating protein 8	BPGAP1, PP610
ARHGAP9		Rho GTPase activating protein 9	10C, RGL1
ARHGAP10		Rho GTPase activating protein 10	GRAF2, PS-GAP, PSGAP, ARHGAP21
ARHGAP11A		Rho GTPase activating protein 11A	GAP (1-12)
ARHGAP11B		Rho GTPase activating protein 11B	B′-T, FAM7B1
ARHGAP12		Rho GTPase activating protein 12	ARHGAP12
ARHGAP13	SRGAP1	SLIT-ROBO Rho GTPase activating protein 1	ARHGAP13
ARHGAP14	SRGAP3	SLIT-ROBO Rho GTPase activating protein 3	ARHGAP14, MEGAP, SRGAP2, WRP
ARHGAP15		Rho GTPase activating protein 15	BM046
ARHGAP17		Rho GTPase activating protein 17	MST066, MST110, MSTP038, MSTP066, MSTP110, NADRIN, PP367, PP4534, RICH-1, RICH1, WBP15
ARHGAP18		Rho GTPase activating protein 18	MacGAP, SENEX, bA307O14.2
ARHGAP19		Rho GTPase activating protein 19	ARHGAP19
ARHGAP20		Rho GTPase activating protein 20	RARHOGAP
ARHGAP21		Rho GTPase activating protein 21	ARHGAP10
ARHGAP22		Rho GTPase activating protein 22	RhoGAP2, RhoGap22
ARHGAP23		Rho GTPase activating protein 23	ARHGAP23
ARHGAP24		Rho GTPase activating protein 24	FILGAP, RC-GAP72, RCGAP72, p73, p73RhoGAP
ARHGAP25		Rho GTPase activating protein 25	HEL-S-308, KAIA0053
ARHGAP26		Rho GTPase activating protein 26	GRAF, GRAF1, OPHN1L, OPHN1L1
ARHGAP27		Rho GTPase activating protein 27	CAMGAP1, PP905, SH3D20, SH3P20
ARHGAP28		Rho GTPase activating protein 28	ARHGAP28
ARHGAP29		Rho GTPase activating protein 29	PARG1
ARHGAP30		Rho GTPase activating protein 30	ARHGAP30
ARHGAP31		Rho GTPase activating protein 31	AOS1, CDGAP
ARHGAP32		Rho GTPase activating protein 32	GC-GAP, GRIT, PX-RICS, RICS, p200RhoGAP, p250GAP
ARHGAP33		Rho GTPase activating protein 33	NOMA-GAP, SNX26, TCGAP
ARHGAP34	SRGAP2	SLIT-ROBO Rho GTPase activating protein 2	ARHGAP34, FNBP2, SRGAP2A, SRGAP3
ARHGAP35		Rho GTPase activating protein 35	GRF-1, GRLF1, P190-A, P190A, p190ARhoGAP, p190RhoGAP
ARHGAP36		Rho GTPase activating protein 36	ARHGAP36
ARHGAP37	STARD13	StAR-related lipid transfer (START) domain containing 13	ARHGAP37, DLC2, GT650, LINC00464
ARHGAP38	STARD8	StAR-related lipid transfer (START) domain containing 8	ARHGAP38, DLC3, STARTGAP3
ARHGAP39		Rho GTPase activating protein 39	CrGAP, Vilse
ARHGAP40		Rho GTPase activating protein 40	C20orf95, dJ1100H13.4
ARHGAP41	OPHN1	oligophrenin 1	ARHGAP41, MRX60, OPN1
ARHGAP42		Rho GTPase activating protein 42	GRAF3
ARHGAP43	SH3BP1	SH3-domain binding protein 1	ARHGAP43
ARHGAP44		Rho GTPase activating protein 44	NPC-A-10, RICH2
ARHGAP45	HMHA1	histocompatibility (minor) HA-1	ARHGAP45, HA-1, HLA-HA1
ARHGAP46	GMIP	GEM interacting protein	ARHGAP46
ARHGAP47	TAGAP	T-cell activation RhoGTPase activating protein	ARHGAP47, FKSG15, IDDM21, TAGAP1
ARHGAP48	FAM13A	family with sequence similarity 13, member A	ARHGAP48, FAM13A1
ARHGAP49	FAM13B	family with sequence similarity 13, member B	ARHGAP49, C5orf5, FAM13B1, KHCHP, N61

Overall, ARHGAP genes harbored a 10% mutation level in BCa according to the analysis of cBioPortal (Figure 1A). The expression of ARHGAP family genes was analyzed in several cancer types in Oncomine, and a relatively lower ARHGAP gene expression was observed in BCa (Supplementary Figure 1A). Moreover, we found that higher expression of ARHGAP mainly predicted unpreferable prognosis in BCa. We further attempted to ascertain the BCa prognosis-related members of ARHGAP family genes and identified that ARHGAP5, ARHGAP8, ARHGAP11A, ARHGAP17, ARHGAP24, ARHGAP37 (STARD13), and ARHGAP38 (STARD8) correlated with either or both RFS and OS in BCa (Figure 1B and Supplementary Figure 1B).

### Correlation and Functional Enrichment Analysis Reveals Rho-GTPase-Activating Proteins Family Genes Correlate With Immune-Related Biological Processes in BC Patients

**FIGURE 2:**
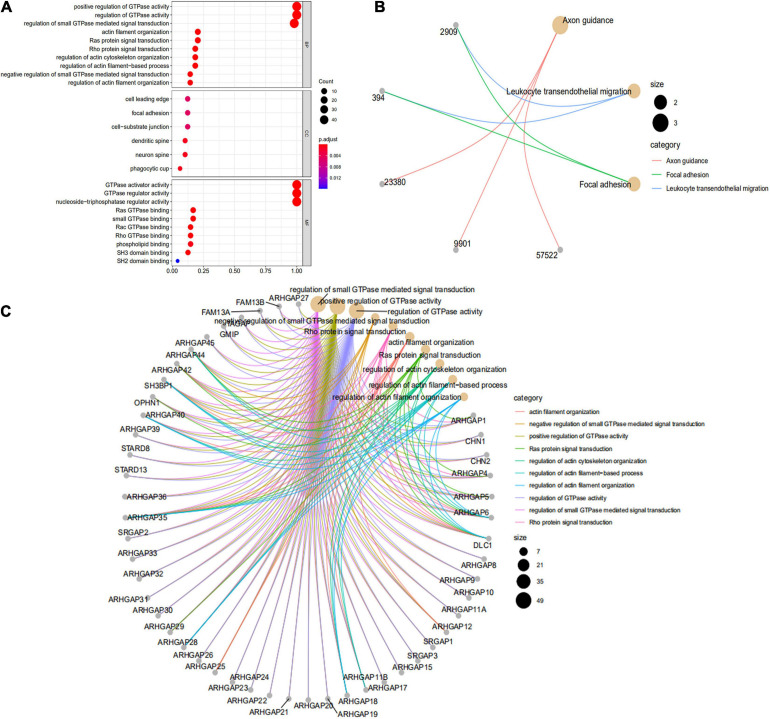
Functional enrichment analysis of the ARHGAP family genes in BCa. **(A)** Top 10 functional enrichment of biological process (BP), cellular component (CC), and molecular function (MF) of the Kyoto Encyclopedia of Genes and Genomes (KEGG) pathway of the ARHGAP family genes in BCa. **(B)** The KEGG pathway analysis of the ARHGAP family genes in The Cancer Genome Atlas (TCGA) cohorts, *p* < 0.05. **(C)** Top 10 enrichment of the Gene Ontology (GO) pathway enrichment.

**FIGURE 3:**
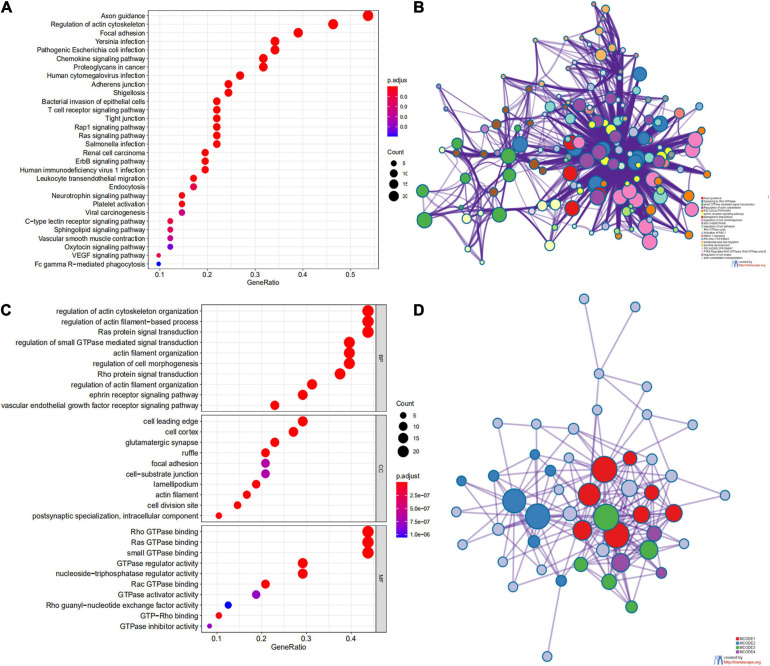
Network and potent construction analysis of 50 ARHGAP family neighboring genes in BCa. **(A)** KEGG pathway analysis of 50 most neighboring correlated genes, *p* < 0.05. **(B)** GO analysis of 50 most neighboring correlated genes, *p* < 0.05. **(C)** Nodes contain differential genes, which are typically close to each other are displayed with the same color. Terms with a similarity > 0.3 are connected by edges. **(D)** Protein–protein interaction network and Molecular Complex Detection (MCODE) components identified of the hub correlating genes.

We first identified the correlation of the ARHGAP family genes in BC patients’ expression levels. As Supplementary Figure 2A showed, colors in blue or red revealed positive or negative correlation, respectively. For example, ARHGAP37 (STARD13) and coexpression with ARHGAP38 (STARD8), positively correlated with ARHGAP31 and ARHGAP24, meaning these ARHGAP genes may exert a synergism biological effect. Then we explored the coexpression genes of the ARHGAP family genes by GeneMANIA (Supplementary Figure 2B). We also identified 50 relevant altered genes by STRING database and constructed PPI network (Supplementary Figure 2C). GO and KEGG functional enrichment analysis were applied to explore the functional process and novel pathways of the ARHGAP family genes. Figure 2A showed 10 most relevant functional processes of ARHGAP family genes in biological process (BP), cellular component (CC), and molecular function (MF), respectively, most of which enriched as regulating GTPase activity and downstream actin organization. As shown in Figure 2B, axon guidance, focal adhesion, and leukocyte transendothelial migration were associated with the ARHGAP family genes. Regulation of small GTPase-mediated signal transduction, positive regulation of GTPase activity, regelation of GTPase activity, etc., were the three most according functional processes (Figure 2C). To further distinguish the correlation of reciprocal genes of the ARHGAP family, we applied GO and KEGG analysis of 50 altered neighboring genes identified by STRING (Figures 3A,B). As we expected, focal adhesion, chemokine signaling pathway, and T-cell receptor signaling pathway were enriched. Then the protein–protein interaction enrichment analysis network was constructed by Metascape following the previous manuscript. As shown in Figure 3C, the subsets that contain the close genes of enriched terms have been selected and presented as a network plot. Besides, we applied Molecular Complex Detection (MCODE) algorithm to identify hub genes among the correlating genes. Pathways and functional enrichment analysis were performed by each MCODE component independently. Each three most significant pathways of MCODE are shown in Supplementary Table 1. All results above implied an association between the ARHGAP family genes and tumorigenesis, migration, and tumor-immunologic microenvironment. ARHGAP regulates GTPase-mediated cell cytoskeleton organization including actin or filament-related focal adhesion and cell motility, which is crucial for immune cells, especially tumor-infiltrating cells for transendothelial migration from the bloodstream to participate in the tumor-immunologic microenvironment. As focal adhesion, leukocyte transendothelial migration from the ARHGAP family genes and focal adhesion, the chemokine signaling pathway and T-cell receptor signaling pathway were enriched from the ARHGAP family genes, we made the hypothesis that the ARHGAP genes regulate immune-related tumor microenvironment in BCa (Figure 3D).

### Expression of Prognosis-Related Rho-GTPase-Activating Proteins Family Genes Correlated With Immune Cell Infiltration in Bladder Cancer

**FIGURE 4:**
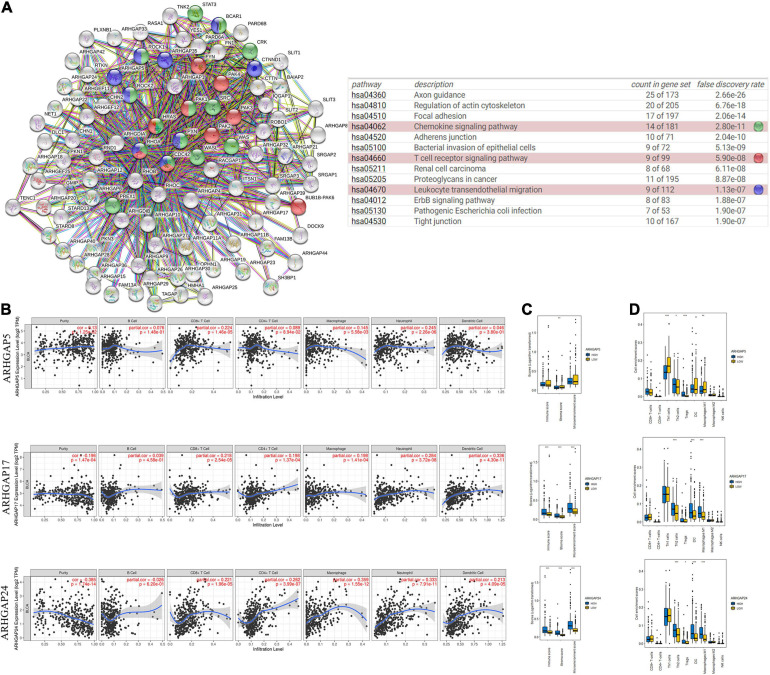
Correlations between prognosis-related ARHGAP family gene-associated immune cell infiltration. **(A)** Immune-related ARHGAP family gene protein–protein interaction (PPI) network was constructed in STRING. **(B)** Immune infiltration correlation with ARHGAP5, ARHGAP17, and ARHGAP24 in TCGA-BCa samples were carried out by TIMER. **(C)** Boxplots were used to visualize ARHGAP family-associated certain cell type enrichment scores and logarithm-transformed immune scores, stroma scores, and microenvironment scores of different groups through xCell-analyzed TCGA samples in BCa. **(D)** ARHGAP family-associated infiltration of CD8+ T cells, CD4+ T cells, type 1 T-helper (Th1) cells, type 2 T-helper (Th2) cells, natural killer (NK) cells, Treg cells, M1 macrophages, M2 macrophages, and dendritic cell were analyzed through xCell-analyzed TCGA samples in BCa. Grouping was done according to the expression level of prognosis-related ARHGAP family gene (*p < 0.05; **p < 0.01; ***p < 0.001; ns, not statistically significant, Wilcoxon signed rank test).

Ras homolog family member A (RhoA) and CDC42 are key downstream components of ARHGAP correlating with leukocyte transendothelial, chemokine signaling pathway, and T-cell receptor signaling pathway in enriched ARHGAP family genes PPI network, indicating that the ARHGAP family genes are involved in several immune pathways in immune-related tumor microenvironment in BCa (Figure 4A). As such, we next explored the relationship between prognosis-related ARHGAP family gene expression and the degree of immune cell infiltration in BCa using xCell and TIMER to analyze the tissue-infiltrating cell-type abundance from bulk RNA-seq data in TCGA database. First, all prognosis-related ARHGAP family gene correlates with tumor purity (Figure 4B and Supplementary Figure 3A). Interestingly, the ARHGAP family genes correlated with immune cell infiltration in different levels. ARHGAP5 and ARHGAP11A showed no significant correlation with immune infiltration score, while ARHGAP8, ARHGAP17, ARHGAP24, ARHGAP37 (STARD13), and ARHGAP38 (STARD8) significantly correlated with immune infiltration score (Figure 4C and Supplementary Figure 3B). For example, a higher ARHGAP5 correlated with lower Th1/Th2 cell ratio, higher Treg cell, and lower M1 macrophage infiltration, indicating a relatively tumor-promoting microenvironment. A higher ARHGAP8 correlated both with lower Th1/Th2 cell ratio and lower M1 macrophage infiltration, which indicated a tumor-promoting microenvironment and lower DC cell infiltration, which contributed to a tumor suppression microenvironment. Lower Th1/Th2 cell ratio, higher DC cell infiltration, and higher Treg cell infiltration were observed in ARHGAP17, ARHGAP24, and ARHGAP37 (STARD13), indicating a tumor-promoting microenvironment (Figures 4B,D, and Supplementary Figure 3C). We further explored the correspondence between the ARHGAP family gene expression and the type of immune infiltration based on immuno-related markers in BCa, especially Tfh cells, Th17 cells, and exhausted T cells in addition to our previous analysis. Besides further validation of the infiltration of monocyte, TAM, M1 macrophage, M2 macrophage, neutrophils, NK cell, DC, Th1, and Th2 cell in prognosis-related ARHGAP family genes in BCa, we further found that higher ARHGAP17, ARHGAP24, ARHGAP37 (STARD13), andARHGAP38 (STARD8) expression positively correlated with T-cell exhaustion markers: PD-1, CTLA4, TIM3, GZMB, and LAG3 (Damo and Joshi, 2019), while ARHGAP5 and ARHGAP8 showed negative or absence of correlation with T-cell exhaustion markers (Table 2). CD8 + T cells exert antitumor activity through regulation of Th1/Th2 cells, Treg cells, DC cells, and macrophages (Xiao et al., 2020) and higher T-cell exhaustion markers indicating a dysfunction state of CD8 + T-cell-related tumor-suppressing microenvironment. In conclusion, through comprehensive analysis of immune cell infiltration in BCa, we identified that the ARHGAP family genes correlate with a tumor-promoting microenvironment.

**TABLE 2:** Correspondence between ARHGAP family gene expression and type of immune infiltration based on immuno-related markers in BCa.

Description	Markers	ARHGAP5				ARHGAP8				ARHGAP11a				ARHGAP17				ARHGAP24				STARD13				STARD8			
								
		None		Purity		None		Purity		None		Purity		None		Purity		None		Purity		None		Purity		None		Purity	
																
		Cor	P	Cor	P	Cor	P	Cor	P	Cor	P	Cor	P	Cor	P	Cor	P	Cor	P	Cor	P	Cor	P	Cor	P	Cor	P	Cor	P
																													
B cell	CD19	–0.124	**1.23E-02**	–0.069	1.86E-01	–0.037	4.58E-01	0.022	6.69E-01	–0.084	8.86E-02	–0.046	3.82E-01	0.248	**3.93E-07**	0.168	**1.25E-03**	0.33	**8.35E-12**	0.178	**6.12E-04**	0.348	**4.61E-13**	0.183	**4.14E-04**	0.435	**2.98E-20**	0.274	**8.94E-08**
	CD79A	–0.074	1.38E-01	–0.015	7.80E-01	–0.054	2.75E-01	0.014	7.85E-01	–0.13	**8.81E-03**	–0.111	**3.34E-02**	0.188	**1.38E-04**	0.065	2.11E-01	0.354	**1.84E-13**	0.182	**4.48E-04**	0.39	**3.11E-16**	0.197	**1.37E-04**	0.471	**6.22E-24**	0.282	**3.53E-08**
T cell(general)	CD2	–0.07	8.81E+01	0.088	9.37E-02	–0.193	**8.92E-05**	–0.137	**8.41E-03**	0.022	6.52E-01	0.078	1.34E-01	0.248	**3.77E-07**	0.134	**9.90E-03**	0.338	**2.46E-12**	0.133	**1.05E-02**	0.391	**8.84E-18**	0.201	**1.01E-04**	0.485	**0.00E+00**	0.27	**1.49E-07**
	CD3D	–0.075	1.31E-01	–0.013	8.05E-01	–0.159	**1.23E-03**	–0.098	5.92E-02	–0.05	3.11E-01	–0.033	5.31E-01	0.164	**8.66E-04**	0.031	5.59E-01	0.282	**6.63E-09**	0.087	9.68E-02	0.345	**8.10E-13**	0.156	**2.71E-03**	0.425	**2.65E-19**	0.206	**7.04E-05**
	CD3E	–0.005	9.20E-01	0.086	1.00E-01	–0.196	**6.45E-05**	–0.137	**8.57E-03**	0.007	8.86E-01	0.063	2.29E-01	0.245	**5.30E-07**	0.121	**2.04E-02**	0.365	**2.87E-14**	0.16	**2.02E-03**	0.418	**0.00E+00**	0.228	**1.02E-05**	0.499	**0.00E+00**	0.277	**6.49E-08**
CD8+ T cell	CD8A	0.011	8.21E-01	0.091	7.99E-02	–0.174	**4.05E-04**	–0.109	**3.66E-02**	0.15	**2.35E-08**	0.217	**2.58E-05**	0.303	**4.25E-10**	0.214	**3.44E-05**	0.315	**7.70E-11**	0.146	**5.01E-03**	0.354	**2.33E-13**	0.189	**2.69E-04**	0.442	**0.00E+00**	0.246	**1.75E-06**
	CD8B	0.006	8.97E-01	0.057	2.74E-01	–0.175	**3.76E-04**	–0.118	**2.41E-02**	0.118	**1.66E-02**	0.161	**1.92E-03**	0.298	**8.15E-10**	0.234	**5.55E-06**	0.25	**3.00E-07**	0.133	**1.05E-02**	0.283	**5.81E-09**	0.152	**3.45E-03**	0.402	**2.70E-17**	0.275	**8.55E-08**
Monocyte	CD86	–0.008	8.73R-01	0.084	1.07E-01	–0.302	**4.79E-10**	–0.267	**2.00E-07**	0.136	**5.96E-03**	0.248	**1.44E-06**	0.344	**8.73E-13**	0.258	**5.35E-07**	0.505	**8.54E-28**	0.355	**2.39E-12**	0.477	**0.00E+00**	0.311	**1.14E-09**	0.562	**0.00E+00**	0.38	**4.04E-14**
	CSF1R	–0.011	8.21E-01	0.075	1.52E-01	–0.265	**5.22E-08**	–0.217	**2.57E-05**	0.039	4.30E-01	0.11	**3.56E-02**	0.364	**3.34E-14**	0.288	**1.81E-08**	0.533	**2.77E-31**	0.39	**7.73E-15**	0.583	**0.00E+00**	0.462	**7.31E-21**	0.669	**0.00E+00**	0.54	**3.07E-29**
TAM	CCL2	–0.097	5.04E-02	–0.028	5.87E-01	–0.067	1.76E-01	0.013	8.02E-01	–0.009	8.63E-01	0.068	1.96E-01	0.381	**1.43E-15**	0.31	**1.24E-09**	0.473	**3.89E-24**	0.315	**6.79E-10**	0.526	**0.00E+00**	0.388	**1.20E-14**	0.601	**0.00E+00**	0.445	**2.55E-19**
	CD68	0.114	**2.17E-02**	0.17	**1.07E-03**	–0.419	**9.17E-19**	–0.392	**6.25E-15**	0.155	**1.72E-03**	0.218	**2.41E-05**	0.185	**1.65E-04**	0.074	1.55E-01	0.365	**2.73E-14**	0.232	**7.16E-06**	0.346	**8.28E-13**	0.197	**1.39E-04**	0.421	**0.00E+00**	0.258	**5.19E-07**
	IL10	–0.064	1.97E+00	–0.004	9.42E-01	–0.137	**5.43E-03**	–0.06	2.52E-01	0.087	7.82E-02	0.177	**6.46E-04**	0.362	**4.70E-14**	0.284	**2.81E-08**	0.533	**2.61E-31**	0.417	**6.92E-17**	0.569	**2.16E-36**	0.457	**2.23E-20**	0.617	**4.48E-44**	0.473	**6.56E-22**
M1 Macrophage	INOS(NOS2)	0.132	**7.79E-03**	0.159	**2.16E-03**	–0.086	8.12E-02	–0.043	4.06E-01	0.117	**1.84E-02**	0.151	**3.64E-03**	0.198	**5.59E-05**	0.181	**4.77E-04**	0.224	**5.03E-06**	0.176	**6.94E-04**	0.186	**1.54E-04**	0.157	**2.58E-03**	0.254	**1.97E-07**	0.195	**1.74E-04**
	IRF5	–0.059	2.32E-01	–0.08	1.26E-01	0.273	**2.07E-08**	0.264	**2.72E-07**	–0.049	3.25E-01	–0.051	3.25E-01	–0.043	3.83E-01	–0.053	3.14E-01	–0.156	**1.56E-03**	–0.165	**1.47E-03**	–0.093	5.99E-02	–0.103	**4.73E-02**	–0.033	5.06E-01	–0.041	4.33E-01
	COX2(PTGS2)	0.258	**1.37E-07**	0.291	**1.37E-08**	0.077	1.21E-01	0.116	**2.64E-02**	0.033	5.09E-01	0.058	2.66E-01	0.098	**4.76E-02**	0.045	3.90E-01	0.207	**2.40E-05**	0.149	**4.26E-03**	0.351	4.11E+13	0.292	**1.08E-08**	0.125	**1.15E-02**	0.036	4.86E-01
M2 Macrophage	CD163	–0.068	1.70E-01	0.006	9.13E-01	–0.229	**2.95E-06**	–0.169	**1.16E-03**	0.12	**1.55E-02**	0.22	**2.06E-05**	0.385	**7.07E-16**	0.318	**4.13E-10**	0.525	**2.51E-30**	0.38	**4.41E-14**	0.549	**0.00E+00**	0.411	**2.02E-16**	0.677	**0.00E+00**	0.551	**1.36E-30**
	VSIG4	–0.065	1.87E-01	0.007	9.00E-01	–0.247	**4.58E-07**	–0.198	**1.32E-04**	0.087	8.02E-02	0.175	**7.49E-04**	0.351	**2.96E-13**	0.28	**4.65E-08**	0.52	**1.32E-29**	0.374	**1.12E-13**	0.533	**0.00E+00**	0.396	**2.99E-15**	0.631	**0.00E+00**	0.487	**2.65E-23**
	MS4A4A	–0.072	1.46E-01	0.003	9.52R-01	–0.258	**1.31E-07**	–0.213	**3.67E-05**	0.098	**4.81E-02**	0.199	**1.25E-04**	0.345	**7.74E-13**	0.266	**2.32E-07**	0.545	**5.85E-33**	0.411	**2.13E-16**	0.546	**0.00E+00**	0.416	**8.41E-17**	0.677	**0.00E+00**	0.545	**7.32E-30**
Neutrophils	CD66b(CEACAM8)	0.102	**3.93E-02**	0.06	2.49E-01	–0.047	3.41E-01	–0.071	1.76E-01	0.091	6.75E-02	0.096	6.51E-02	0.072	1.48E-01	0.081	1.20E-01	0.114	**2.17E-02**	0.124	**1.71E-02**	0.057	2.53E-01	0.083	1.13E-01	0.037	4.57E-01	0.049	3.50E-01
	CD11b(ITGAM)	–0.058	2.46E-01	0.007	8.93E-01	–0.164	**8.87E-04**	–0.088	9.04E-02	0.073	1.39E-01	0.149	**4.16E-03**	0.395	**1.16E-16**	0.324	**1.86E-10**	0.476	**1.83E-24**	0.327	**1.31E-10**	0.491	**0.00E+00**	0.339	**2.36E-11**	0.618	**0.00E+00**	0.464	**4.76E-21**
	CCR7	–0.184	**1.93E-04**	–0.146	**4.87E-03**	0.246	**4.70E-07**	0.266	**2.13E-07**	–0.17	**5.69E-04**	–0.166	**1.39E-03**	0.005	9.19E-01	–0.046	3.80E-01	0.035	4.78E-01	–0.035	5.07E-01	0.125	**1.15E-02**	0.031	5.56E-01	0.167	**6.98E-04**	0.104	**4.61E-02**
Natural killer cell	KIR2DL1	–0.072	1.48E-01	–0.055	2.96E-01	–0.108	**2.95E-02**	–0.055	2.92E-01	0.068	1.70E-01	0.077	1.39E-01	0.147	**3.00E-03**	0.063	2.26E-01	0.171	**5.36E-04**	0.042	4.21E-01	0.153	**1.97E-03**	0.033	5.30E-01	0.249	3.40E-07	0.108	**3.80E-02**
	KIR2DL3	–0.049	3.23E-01	0.001	9.87E-01	–0.183	**1.99E-04**	–0.124	**1.73E-02**	0.112	**2.37E-02**	0.137	**8.37E-03**	0.273	**2.15E-08**	0.189	**2.71E-04**	0.198	**5.84E-05**	0.047	3.65E-01	0.217	**9.55E-06**	0.078	1.33E-01	0.338	**2.44E-12**	0.174	**7.82E-04**
	KIR2DL4	0.038	4.44E-01	0.1	5.46E-02	–0.223	**5.67E-06**	–0.169	**1.13E-03**	0.24	**9.64E-07**	0.295	**7.91E-09**	0.194	**7.68E-05**	0.122	**1.97E-02**	0.225	**4.26E-06**	0.094	7.19E-02	0.171	**5.35E-04**	0.033	5.29E-01	0.234	**1.81E-06**	0.064	2.19E-01
	KIR3DL1	–0.045	3.60E-01	–0.01	8.52E-01	–0.11	**2.63E-02**	–0.051	3.29E-01	0.043	3.84E-01	0.035	5.05E-01	0.229	**3.05E-06**	0.173	**8.84E-04**	0.154	**1.78E-03**	0.044	4.02E-01	0.21	**1.98E-05**	0.124	**1.74E-02**	0.29	**2.42E-09**	0.191	**2.26E-04**
	KIR3DL2	–0.046	3.57E-01	–0.009	8.66E-01	–0.125	**1.15E-02**	–0.068	1.92E-01	0.104	**3.51E-02**	0.129	**1.31E-02**	0.143	**3.80E-03**	0.061	2.46E-01	0.165	**8.23E-04**	0.028	5.97E-01	0.177	**3.25E-04**	0.046	3.79E-01	0.263	**6.87E-08**	0.11	**3.42E-02**
	KIR3DL3	0.002	9.69E-01	0.012	8.21E-01	–0.117	**1.80E-02**	–0.094	7.16E-02	0.121	**1.44E-02**	0.12	**2.10E-02**	0.027	5.87E-01	–0.01	8.44E-01	0.044	3.76E-01	–0.01	8.54E-01	0.031	5.26E-01	–0.021	6.94E-01	0.035	4.85E-01	–0.038	4.70E-01
	KIR2DS4	–0.065	1.93E-01	–0.029	5.77E-01	–0.161	**1.12E-03**	–0.109	**3.71E-02**	0.13	**8.62E-03**	0.137	**8.29E-03**	0.176	**3.68E-04**	0.094	7.09E-02	0.126	**1.08E-02**	–0.004	9.39E-01	0.15	**2.44E-03**	0.032	5.44E-01	0.217	**9.83E-06**	0.068	1.94E-01
Dendritic cell	HLA-DPB1	–0.081	1.01E-01	–0.015	7.70E-01	–0.246	**4.62E-07**	–0.197	**1.38E-04**	–0.013	7.93E-01	0.036	4.95E-01	0.306	**2.91E-10**	0.21	**4.81E-05**	0.374	**5.13E-15**	0.177	**6.69E-04**	0.46	**0.00E+00**	0.281	**4.27E-08**	0.556	**0.00E+00**	0.371	**1.80E-13**
	HLA-DQB1	–0.011	8.27E-01	0.079	1.29E-01	–0.222	**6.15E-06**	–0.173	**8.41E-04**	0.015	7.69E-01	0.063	2.30E-01	0.288	**2.98E-09**	0.2	**1.13E-04**	0.33	**7.54E-12**	0.154	**3.07E-03**	0.395	**1.14E-16**	0.231	**7.51E-06**	0.46	**8.45E-23**	0.253	**8.98E-07**
	HLA-DRA	0.02	6.93E-01	0.111	3.33E-02	–0.252	**2.51E-07**	–0.197	**1.42E-04**	0.062	2.11E-01	0.117	**2.50E-02**	0.299	**6.93E-10**	0.207	**6.31E-05**	0.351	**3.06E-13**	0.168	**1.22E-03**	0.438	**0.00E+00**	0.272	**1.13E-07**	0.513	**0.00E+00**	0.324	**2.01E-10**
	HLA-DPA1	0.007	8.95E-01	0.09	8.44E-02	–0.251	**2.79E-07**	–0.2	**1.12E-04**	0.045	3.67E-01	0.101	5.34E-02	0.315	**7.56E-11**	0.231	**7.93E-06**	0.374	**5.33E-15**	0.199	**1.17E-04**	0.456	**2.24E-22**	0.298	**5.53E-09**	0.539	**3.74E-32**	0.365	**5.11E-13**
	BDCA-1(CD1C)	0.099	**4.53E-02**	0.151	**3.75E-03**	–0.229	**2.86E-06**	–0.189	**2.67E-04**	–0.13	**8.62E-03**	–0.114	**2.91E-02**	0.092	6.20E-02	0.016	7.59E-01	0.341	**1.45E-12**	0.213	**3.87E-05**	0.361	**5.60E-14**	0.232	**6.93E-06**	0.386	**6.58E-16**	0.255	**7.28E-07**
	BDCA-4(NRP1)	0.172	**4.78E-04**	0.216	**2.82E-05**	–0.225	**4.40E-06**	–0.182	**4.59E-04**	0.294	**1.76E-09**	0.357	**1.62E-12**	0.397	**8.04E-17**	0.334	**4.91E-11**	0.608	**1.22E-42**	0.533	**1.92E-28**	0.569	**0.00E+00**	0.532	**3.08E-28**	0.661	**0.00E+00**	0.592	**3.21E-36**
	CD11c(ITGAX)	–0.082	**9.93E-02**	–0.009	8.64E-01	–0.18	**2.51E-04**	–0.107	**3.93E-02**	0.1	**4.34E-02**	0.205	**7.26E-05**	0.392	**1.79E-16**	0.329	**9.40E-11**	0.52	**1.38E-29**	0.365	**4.61E-13**	0.484	**0.00E+00**	0.309	**1.40E-09**	0.594	**0.00E+00**	0.405	**6.05E-16**
Th1	T-bet(TBX21)	–0.035	4.77E-01	0.027	5.99E-01	–0.187	**1.49E-04**	–0.127	**1.50E-02**	0.091	6.71E-02	0.143	**6.01E-03**	0.297	**9.41E-10**	0.191	**2.36E-04**	0.342	**1.19E-12**	0.154	**3.00E-03**	0.347	**5.35E-13**	0.161	**1.98E-03**	0.477	**1.47E-24**	0.273	**1.05E-07**
	STAT4	0.072	1.46E-01	0.171	**1.02E-03**	–0.264	**6.07E-08**	–0.229	**9.20E-06**	0.085	8.62E-02	0.161	**2.00E-03**	0.303	**4.03E-10**	0.215	**3.12E-05**	0.434	**3.93E-20**	0.268	**1.87E-07**	0.426	**1.90E-19**	0.266	**2.12E-07**	0.44	**1.00E-20**	0.226	**1.17E-05**
	STAT1	0.273	**2.39E-08**	0.362	**7.20E-13**	–0.192	**9.51E-05**	–0.129	**1.32E-02**	0.348	**6.59E-18**	0.406	**4.70E-16**	0.294	**1.36E-09**	0.214	**3.52E-05**	0.292	**1.86E-09**	0.151	**3.60E-03**	0.294	**1.65E-09**	0.159	**2.20E-03**	0.302	**5.56E-10**	0.13	**1.25E-02**
	IFNG	–0.047	3.39E-01	0.01	8.53E-01	–0.124	**1.19E-02**	–0.068	1.91E-01	0.197	**6.25E-05**	0.235	**5.35E-06**	0.229	**3.08E-06**	0.147	**4.78E-03**	0.197	**6.30E-05**	0.053	3.13E-01	0.166	**7.43E-04**	0.012	8.20E-01	0.246	**4.65E-07**	0.054	3.04E-01
	TNF	0.062	2.13E-01	0.126	**1.57E-02**	–0.065	1.91E-01	–0.032	5.42E-01	0.153	**1.99E-03**	0.18	**5.19E-04**	0.33	**8.27E-12**	0.255	**7.07E-07**	0.225	**4.34E-06**	0.112	**3.16E-02**	0.227	**3.75E-06**	0.115	**2.77E-02**	0.286	**3.87E-09**	0.144	**5.57E-03**
Th2	GATA3	0.001	9.92E-01	–0.03	**5.61E-04**	0.38	**1.86E-15**	0.332	**6.10E-11**	–0.223	**5.95E-06**	–0.264	**2.84E-07**	–0.199	**4.97E-05**	–0.138	**8.17E-03**	–0.384	**9.17E-16**	–0.287	**2.16E-08**	–0.192	**9.57E-05**	–0.062	2.37E-01	–0.307	**2.81E-10**	–0.187	**3.12E-04**
	STAT6	0.319	**5.76E-11**	0.292	**1.11E-08**	0.016	7.50E-01	0	9.95E-01	–0.048	3.32E-01	–0.086	9.80E-02	–0.033	5.06E-01	–0.005	9.19E-01	–0.092	6.45E-02	–0.039	4.61E-01	–0.05	3.17E-01	0.034	5.13E-01	–0.118	**1.74E-02**	–0.05	3.35E-01
	STAT5A	–0.038	4.47E-01	–0.017	7.45E-01	–0.132	**7.39E-03**	–0.084	1.08E-01	–0.035	4.69E-01	–0.007	8.89E-01	0.293	**1.59E-09**	0.235	**4.98E-06**	0.242	**7.72E-07**	0.112	**3.17E-02**	0.319	**5.20E-11**	0.182	**4.57E-04**	0.387	**2.05E-16**	0.268	**1.76E-07**
	IL13	0	9.93E-01	0.038	4.70E-01	–0.117	**1.85E-02**	–0.099	5.88E-02	–0.067	1.74E-01	–0.059	2.62E-01	0.137	**5.43E-03**	0.08	1.26E-01	0.235	**1.54E-06**	0.116	**2.66E-02**	0.221	**6.44E-06**	0.112	**3.24E-02**	0.31	**1.56E-10**	0.199	**1.21E-04**
Tfh	BCL6	0.274	**1.99E-08**	0.258	**5.39E-07**	–0.002	9.66E-01	0.004	9.40E-01	–0.051	3.04E-01	–0.074	1.58E-01	–0.106	**3.23E-02**	–0.085	1.03E-01	–0.019	6.98E-01	–0.002	9.63E-01	–0.036	4.71E-01	0	9.99E-01	–0.088	**7.62E-02**	–0.051	3.28E-01
	IL21	–0.045	3.70E-01	–0.005	9.25E-01	–0.061	2.22E-01	–0.022	6.67E-01	0.138	**5.37E-03**	0.166	**1.39E-03**	0.185	**1.72E-04**	0.147	**4.79E-03**	0.148	**2.78E-03**	0.089	8.67E-02	0.126	**1.06E-02**	0.049	3.46E-01	0.275	**1.71E-08**	0.22	**2.07E-05**
Th17	IL17A	0.064	2.01E-01	0.078	1.37E-01	0.007	8.90E-01	0.001	9.91E-01	–0.038	4.48E-01	–0.032	5.40E-01	–0.099	**4.67E-02**	–0.138	**8.21E-03**	–0.095	5.49E-02	–0.129	**1.31E-02**	–0.058	2.45E-01	–0.089	**8.67E-02**	–0.041	4.07E-01	–0.089	**8.66E-02**
	STAT3	0.457	0.00E+00	0.511	**7.46E-26**	–0.224	**4.73E-06**	–0.172	**9.05E-04**	0.311	**1.67E-10**	0.334	**5.21E-11**	0.366	**2.35E-14**	0.308	**1.66E-09**	0.392	**2.06E-16**	0.289	**1.61E-08**	0.375	**5.17E-15**	0.29	**1.38E-08**	0.32	**4.42E-11**	0.204	**8.13E-05**
Treg	FOXP3	0.124	1.21E-02	0.238	**3.86E-06**	–0.191	**1.04E-04**	–0.141	**6.79E-03**	0.159	**1.33E-03**	0.238	**3.82E-06**	0.339	**2.10E-12**	0.262	**3.37E-07**	0.433	**4.16E-20**	0.31	**1.16E-09**	0.458	**0.00E+00**	0.335	**4.48E-11**	0.543	**0.00E+00**	0.398	**2.19E-15**
	CCR8	0.222	**5.75E-06**	0.346	**8.30E-12**	–0.079	1.10E-01	–0.015	7.77E-01	0.218	**9.15E-06**	0.295	**8.10E-09**	0.3	**6.04E-10**	0.216	**2.87E-05**	0.414	**2.64E-18**	0.298	**5.21E-09**	0.498	**5.81E-27**	0.402	**9.73E-16**	0.539	**4.57E-32**	0.412	**1.63E-16**
	STAT5B	0.43	**0.00E+00**	0.42	**3.42E-17**	–0.076	1.24E-01	–0.069	1.84E-01	0.224	**5.44E-06**	0.236	**4.91E-06**	0.291	**1.98E-09**	0.307	**1.88E-09**	0.325	**1.64E-11**	0.346	**9.25E-12**	0.322	**3.42E-11**	0.341	**1.70E-11**	0.334	**5.39E-12**	0.38	**4.60E-14**
	TGFB1	0.082	9.76E-02	0.092	7.67E-02	–0.195	**7.10E-05**	–0.165	**1.52E-03**	0.034	4.98E-01	0.063	2.30E-01	0.197	**6.26E-05**	0.14	**7.23E-03**	0.286	**4.08E-09**	0.199	**1.22E-04**	0.298	**1.02E-09**	0.211	**4.45E-05**	0.217	**1.07E-05**	0.106	**4.29E-02**
T cell exhaustion	PD-1(PDCD1)	–0.06	2.30E-01	0.012	8.16E-01	–0.162	**1.04E-03**	–0.099	**5.90E-02**	0.072	1.46E-01	0.128	**1.41E-02**	0.295	**1.21E-09**	0.198	**1.28E-04**	0.321	**3.02E-11**	0.137	**8.28E-03**	0.363	**3.65E-14**	0.183	**4.13E-04**	0.469	**1.09E-23**	0.26	**4.28E-07**
	CTLA4	–0.033	5.03E-01	0.061	2.42E-01	–0.151	**2.17E-03**	–0.086	**9.86E-02**	0.087	8.09E-02	0.148	**4.41E-03**	0.276	**1.48E-08**	0.176	**6.93E-04**	0.339	**2.10E-12**	0.165	**1.54E-03**	0.367	**1.72E-14**	0.188	**2.83E-04**	0.436	**2.39E-20**	0.222	**1.73E-05**
	TIM3(HAVCR2)	–0.041	4.09E-01	0.044	3.97E-01	–0.243	**6.76E-07**	–0.186	**3.37E-04**	0.132	**7.83E-03**	0.235	**5.38E-06**	0.35	**3.57E-13**	0.262	**3.26E-07**	0.474	**2.93E-24**	0.305	**2.28E-09**	0.478	**0.00E+00**	0.308	**1.50E-09**	0.59	**0.00E+00**	0.41	**2.53E-16**
	GZMB	–0.091	6.56E-02	–0.019	7.22E-01	–0.154	**1.85E-03**	–0.075	1.51E-01	0.103	**3.75E-02**	0.17	**1.09E-03**	0.254	**1.96E-07**	0.152	**3.48E-03**	0.302	**5.07E-10**	0.096	6.58E-02	0.304	**3.36E-10**	0.105	**4.47E-02**	0.37	**1.02E-14**	0.124	**1.70E-02**
	LAG3	–0.038	4.48E-01	0.036	4.90E-01	–0.207	**2.42E-05**	–0.151	**3.64E-03**	0.192	1.02E-04	0.27	**1.49E-07**	0.309	**1.87E-10**	0.217	**2.65E-05**	0.333	**5.32E-12**	0.166	**1.38E-03**	0.297	**1.15E-09**	0.113	**2.96E-02**	0.394	**0.00E+00**	0.184	**4.02E-04**

### Knockdown of ARHGAP5, ARHGAP17, and ARHGAP24 Suppressed BC Cell Proliferation, Migration, and Metastasis *in vitro* and *in vivo*

**FIGURE 5:**
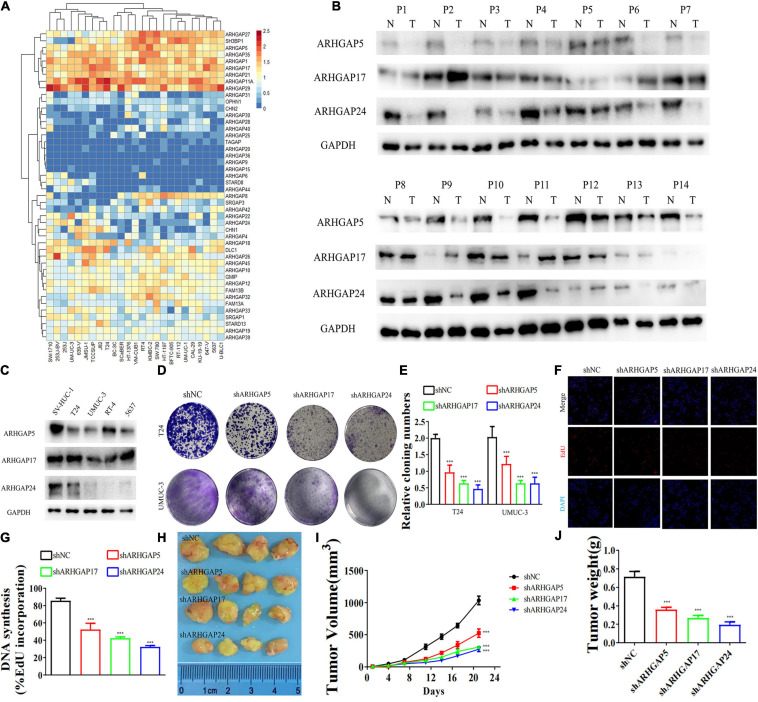
Knockdown of ARHGAP5-, ARHGAP17-, and ARHGAP24-suppressed BC cell proliferation *in vivo* and *in vitro*. **(A)** System analysis of expression of ARHGAP family gene in BCa cancer cell lines in the CCLE database. **(B)** ARHGAP5, ARHGAP17, and ARHGAP24 expression in seven pairs of low-grade BCa and seven pairs of high-grade BCa; GAPDH serves as a loading control. **(C)** ARHGAP5, ARHGAP17, and ARHGAP24 expression in four bladder transitional cell carcinoma cell lines (RT4, UM-UC-3, T24, and 5637) compared with immortalized epithelium cell line (SV-HUC-1). **(D,E)** Cloning formation of T24, UMUC-3, and ARHGAP5, ARHGAP17, and ARHGAP24 knocking down T24, UMUC-3 (***p < 0.001 versus shNC). **(F,G)** 5-Ethynyl-2′-deoxyuridine (EdU) assay of T24 and shARHGAP5-, ARHGAP17-, and ARHGAP24-transfected T24 (***p < 0.001 versus shNC). **(H)** Tumor growth size of subcutaneous T24 tumors transfected with shNC or shARHGAP5, ARHGAP17, and ARHGAP24 21 days after tumor implantation. **(I)** Tumor growth curve of subcutaneous T24 tumors transfected with shNC or shARHGAP5, ARHGAP17, and ARHGAP24 in 21 days of tumor implantation. **(J)** Average weights of tumors from shNC and shARHGAP5, ARHGAP17, and ARHGAP24 are shown in the histogram. Error bars indicate SD.

**FIGURE 6:**
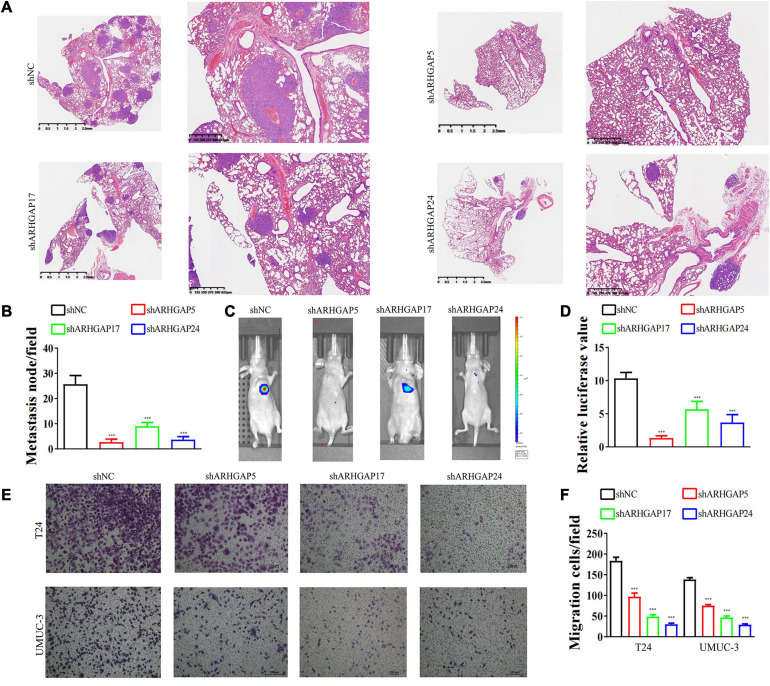
Knockdown of ARHGAP5, ARHGAP17, and ARHGAP24 suppressed the metastasis and migration of BC cell *in vivo* and *in vitro*. **(A,B)** Representative hematoxylin and eosin-stained taken from shNC-T24 and shARHGAP5, ARHGAP17, and ARHGAP24-T24-bearing mice (***p < 0.001 versus shNC). **(C,D)** Bioluminescence imaging in mice T24 metastasis model and knockdown ARHGAP5, ARHGAP17, and ARHGAP24 T24 cell metastasis model (***p < 0.001 versus shNC). **(E,F)** Transwell assay showed that ARHGAP5, ARHGAP17, and ARHGAP24 knockdown hindered migration of T24 and UM-UC-3 (***p < 0.001 versus shNC).

We further began to study the biological role of prognosis-related ARHGAP genes in BCa. Through system analysis of ARHGAP family gene expression in BCa cell lines through CCLE (Barretina et al., 2012; Figure 5A), we selected ARHGAP5, ARHGAP17, and ARHGAP24 for further exploration because of their relatively high expression and immuno-related role in BCa. ARHGAP5 and ARHGAP24 were significantly downregulated in BCa cell lines compared with normal SV-HUC-1 cells and BCa tumor compared with adjacent normal tissue, while ARHGAP17 showed no significant difference (Figures 5B,C and Table 3). We designed three shRNAs targeting ARHGAP5, ARHGAP17, and ARHGAP24, respectively, and the most significant reduction in parental gene expression of shRNA was selected. Cell cloning formation assays showed that knockdown of ARHGAP5, ARHGAP17, and ARHGAP24 significantly inhibited proliferation of T24 and UM-UC-3 (Figures 5D,E). The EdU assay presented similar results based on detecting cellular DNA synthesis (Figures 5F,G). T24 cells transfected with scramble shRNA (sh-NC) and shRNA targeting ARHGAP5, ARHGAP17, and ARHGAP24 were injected into nude mice subcutaneously, and knockdown of these genes significantly reduced BC tumor growth (Figures 5H–J). To verify the metastasis function of the ARHGAP family *in vivo*, we chose the tail vein injection lung metastasis mouse model. The metastatic foci were significantly visible from mice injected with T24 cells in comparison with sh-T24 cells (Figure 6A). Through correspondence with the micropathological results, H&E-stained tissues showed that sh-T24 cells formed up fewer nodule areas in the lungs (Figures 6A,B). Bioluminescence imaging revealed that silencing ARHGAP5, ARHGAP17, and ARHGAP24 reduced the metastasis potential of T24 cells (Figures 6C,D). Transwell assay suggested that ARHGAP5, ARHGAP17, and ARHGAP24 knockdown inhibited migration of T24 and UM-UC-3 (Figures 6E,F). Taken together, our data showed that, in addition to the immuno-related role in BCa, silencing ARHGAP5, ARHGAP17, and ARHGAP24 suppressed BC cell proliferation, migration, and metastasis.

**TABLE 3:** Clinicopathological features in 14 pairs of bladder cancer frozen in liquid nitrogen for analysis of ARHGAP isoforms.

Characteristics	No. (%)
**Gender**	
Male	10 (71.4)
Female	4 (28.6)
**Age**	
< 65	9 (64.3)
≥ 65	5 (35.7)
**Tumor size**	
< 3 cm	6 (42.9)
≥ 3 cm	8 (57.1)
**Clinical stage**	
Ta-T1	8 (57.1)
T2-T4	6 (42.9)
**Grade**	
Low	7 (50.0)
High	7 (50.0)
**Lymphatic metastasis**	
Yes	3 (21.4)
No	11 (78.6)
**Muscle invasion**	
NMIBC	8 (57.1)
MIBC	6 (42.9)
Total	14

### Discovering Underlying Molecular Mechanism of ARHGAP5, ARHGAP17, and ARHGAP24 in Bladder Cancer

**FIGURE 7:**
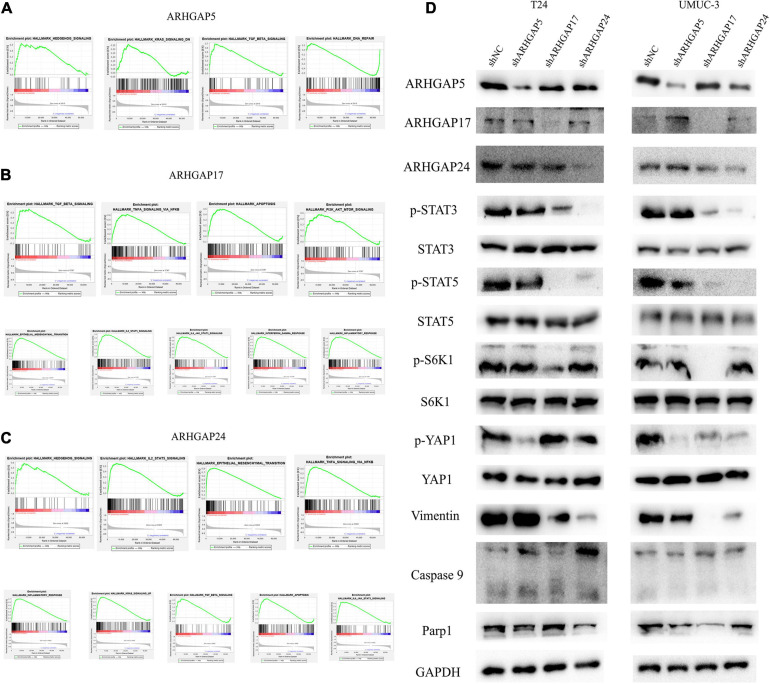
Molecular mechanism of representative ARHGAP family genes in BC biological process. Gene set enrichment analysis (GSEA) plot of **(A)** ARHGAP5, **(B)** ARHGAP17, and **(C)** ARHGAP24, *p* < 0.05. False discovery rate (FDR) < 0.25. **(D)** Western bolt analysis of p-STAT3, STAT3, p-STAT5, STAT5, p-S6K1, S6K1, p-YAP1, YAP1, vimentin, caspase 9, and Parp1 in T24, UMUC-3, and ARHGAP5, ARHGAP17, and ARHGAP24 knocking down T24, UMUC-3.

We further performed gene set enrichment analysis (GSEA) of ARHGAP5, ARHGAP17, and ARHGAP24 in BCa based on TCGA database to analyze downstream pathway. As for immune-related pathway, ARHGAP5 (Figure 7A and Supplementary Table 2) only correlates with TGF-β signaling, while ARHGAP17 (Figure 7B, Supplementary Table 3) and ARHGAP24 (Figure 7C, Supplementary Table 4) correlate with TGF-β, TNF-α, IL-2/STAT5, IL-6/JAK/STAT3, and the inflammatory response pathway, which, in line with our previous result, showed an immune-related role of ARHGAP17 and ARHGAP24 rather than ARHGAP5 (Figure 4C). Also, we observed that ARHGAP5 correlates with HALLMARK_PI3K_AKT_MTOR_SIGNALING, HALLMARK _KRAS_SIGNALING, and HALLMARK_HEDGEHOG_SIGNALING, while ARHGAP17 correlates with HALLMARK_APOPTOSIS, HALLMARK_EPITHELIAL_MESENCHYMAL_TRANSITION, HALLMARK_PI3K_AKT_MTOR_SIGNALING and HALLMARK_ KRAS_SIGNALING, and ARHGAP24 correlates with HALLMARK_KRAS_SIGNALING, HALLMARK_ HEDGEHOG_SIGNALING, HALLMARK_ EPITHELIAL_ MESENCHYMAL_ TRANSITION, and HALLMARK_ APOPTOSIS. We further validated the key pathway indicator protein *via* particular knocking down of ARHGAP5, ARHGAP17, and ARHGAP24 in T24 and UMUC-3. Significant downregulation of p-s6k was observed in sh-ARHGAP17-T24 or UMUC-3, not in ARHGAP5 or ARHGAP24 knocking down cells. Inhibition of vimentin was specifically seen after knocking down ARHGAP17 and ARHGAP24, indicating that ARHGAP17 and ARHGAP24 could activate EMT in BCa cells. Activation of YAP was abolished in ARHGAP5 knocking down BCa cells T24 and UMUC-3, while inhibition of YAP was only observed in ARHGAP17, and ARHGAP24 knocked down UMUC-3. No significant cleaved form of PARP1 or caspase 9 was observed in ARHGAP knocked down BCa, indicating little contribution of ARHGAP in cellular apoptosis. Furthermore, significant deficiency of p-STAT3 and p-STAT5 in shARHGAP17 and shARHGAP24 BCa cells were validated according to GSEA enrichment (Figure 7D). GSEA analysis based on TCGA database and validation experiment indicated that ARHGAP5, ARHGAP17, and ARHGAP24, representing the ARHGAP family, showed, to some extent, difference, rather than similarity, in downstream pathway, performing diverse biological activities while serving as the activator of Rho GTPase.

### ARHGAP17 and ARHGAP24 Correlated With CD8+ T Cells and Treg Infiltration and T-Cell Exhaustion Markers in Bladder Cancer Microenvironment

**FIGURE 8:**
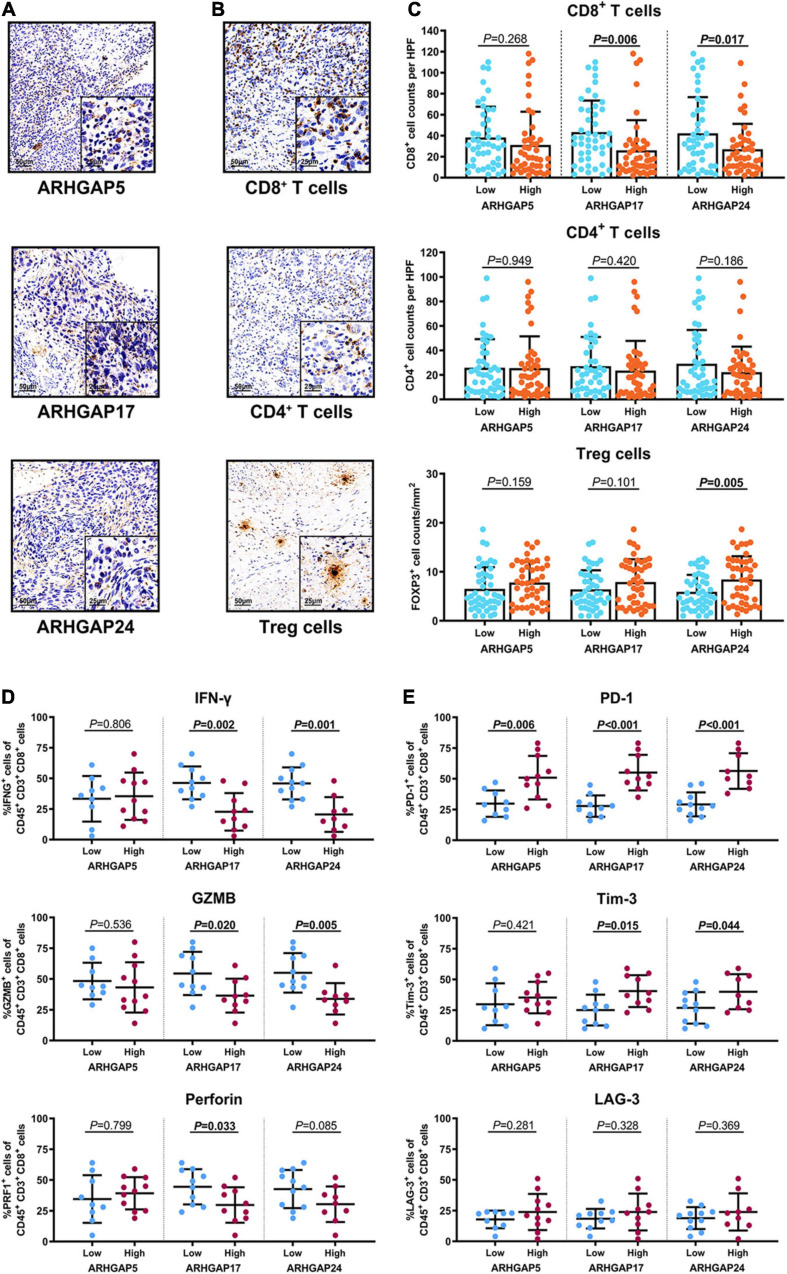
Immunosuppressive tumor microenvironment was associated with ARHGAP family expression. **(A,B)** Representative immunohistochemistry staining of the ARHGAP family [**(A)**; ARHGAP5, ARHGAP17, and ARHGAP24] and immune cells [**(B)**; CD8 + T cells, CD4 + T cells, and Treg cells]. **(C)** Comparison of CD8 + T cell, CD4 + T cell, and Treg cell infiltration in bladder cancer according to ARHGAP5, ARHGAP17, and ARHGAP24 high/low groups. **(D,E)** Comparison of effector molecules [**(D)**; IFN-γ, GZMB, and perforin] and immune checkpoint [**(E)**; PD-1, Tim-3, and LAG-3] in bladder cancer according to ARHGAP5, ARHGAP17, and ARHGAP24 high/low groups.

We have demonstrated that the ARHGAP family genes correlated with an immuno-related tumor-promoting microenvironment through bioinformatic analysis, and our *in vivo* and *in vitro* experiments validated that ARHGAP5, ARHGAP17, and ARHGAP24 promoted the proliferation, migration, and metastasis phenotype of BCa cells. However, these *in silico* results, which indicated ARHGAP expression, correlating with a tumor-supporting microenvironment, need further validation to elucidate the relative contribution of tumor and leukocyte ARHGAP in BCa. We performed immunohistochemistry staining on 90 tissues of TMA (Table 4) and conducted flow cytometry for the tumor-infiltrating CD8 + T cells to explore the association between ARHGAP family and tumor immune microenvironment. ARHGAP5, ARHGAP17, and ARHGAP 24 were detected in bladder cancer tumor sections as well as CD8 + T cells, CD4 + T cells, and Treg cells (Figures 8A,B). We found that decreased CD8 + T cells were infiltrated in ARHGAP17 and ARHGAP 24 high groups than the low ones. As in *in silico* analysis, expression of ARHGAP5, ARHGAP17, and ARHGAP24 shows no correlation with CD4 + T-cell infiltration (Figure 8C). Moreover, the Treg cells showed an increased infiltration in the ARHGAP24 high group. The function of CD8 + T cells were detected through flow cytometry (Table 5 and Supplementary Figure 4). Interestingly, less IFN-γ and GZMB were expressed on CD8 + T cells in high vs. low ARHGAP17 and ARHGAP24 groups, and the CD8 + T cells in the high ARHGAP 17 group expressed declined perforin, which indicate a dampened antitumor function of CD8 + T cells with high ARHGAP17/24 expression (Figure 8D). Moreover, PD-1 was highly expressed on CD8 + T cells in the high ARHGAP5/17/24 groups, and more CD8 + T cells in the high ARHGAP17/24 groups expressed Tim-3 (Figure 8E). These results demonstrated that ARHGAP17/24 may lead to an immunosuppressive tumor microenvironment and an impaired antitumor state of CD8 + T cells in bladder cancer. Considering that higher T-cell exhaustion markers indicating a CD8 + T-cell-related tumor-promoting microenvironment and CD8 + T cells exert antitumor activity through Treg cell regulation (Xiao et al., 2020), our data demonstrated that ARHGAP17 and ARHGAP24 correlated with CD8 + T cells and Treg cell infiltration and function in BCa microenvironment. We noticed that ARHGAP5 showed less correlation with leukocyte infiltration and function. Interestingly, ARHGAP17 and ARHGAP24 influenced immuno-related IL-2/STAT5 and IL-6/JAK/STAT3 pathways, while ARHGAP5 mainly correlated pathways, which have less interaction with immune related environment. Also, in Figure 4C, ARHGAP5 showed no correlation with immuno-microenvironment, while ARHGAP17 and ARHGAP24 correlated with immuno-microenvironment in BCa through xcell analysis. In conclusion, our results indicated that through crosstalk of tumor and leukocyte in BCa, ARHGAP17 and ARHGAP24 correlate with a tumor-promoting microenvironment through regulating CD8 + T cells and Treg infiltration and T cell function.

**TABLE 4:** Clinicopathological features in Tissue Microarray of 90 pairs of bladder cancer.

Characteristics	No. (%)
**Gender**	
Male	81 (90.0)
Female	9 (10.0)
**Age**	
< 65	56 (62.2)
≥ 65	34 (37.8)
**Tumor size**	
< 3 cm	38 (42.2)
≥ 3 cm	52 (57.8)
**Clinical stage**	
Ta-T1	44 (48.9)
T2-T4	46 (51.1)
**Grade**	
Low	51 (56.7)
High	39 (43.3)
**Lymphatic metastasis**	
Yes	22 (24.4)
No	68 (75.6)
**Muscle invasion**	
NMIBC	61 (67.8)
MIBC	29 (32.2)
Total	90

**TABLE 5:** Clinicopathological features of 20 fresh bladder cancer for Flow cytometry analysis.

Characteristics	No. (%)
**Gender**	
Male	14 (70.0)
Female	6 (20.0)
**Age**	
< 65	13 (65.0)
≥ 65	7 (35.0)
**Tumor size**	
< 3 cm	10 (50.0)
≥ 3 cm	10 (50.0)
**Clinical stage**	
Ta-T1	12 (60.0)
T2-T4	8 (40.0)
**Grade**	
Low	11 (55.0)
High	9 (45.0)
**Lymphatic metastasis**	
Yes	4 (20.0)
No	16 (80.0)
**Muscle invasion**	
NMIBC	12 (60.0)
MIBC	8 (40.0)
Total	20

### Rho-GTPase-Activating Proteins Maintain Malignancy-Related Cellular Mechanical Properties of Bladder Cancer

**FIGURE 9:**
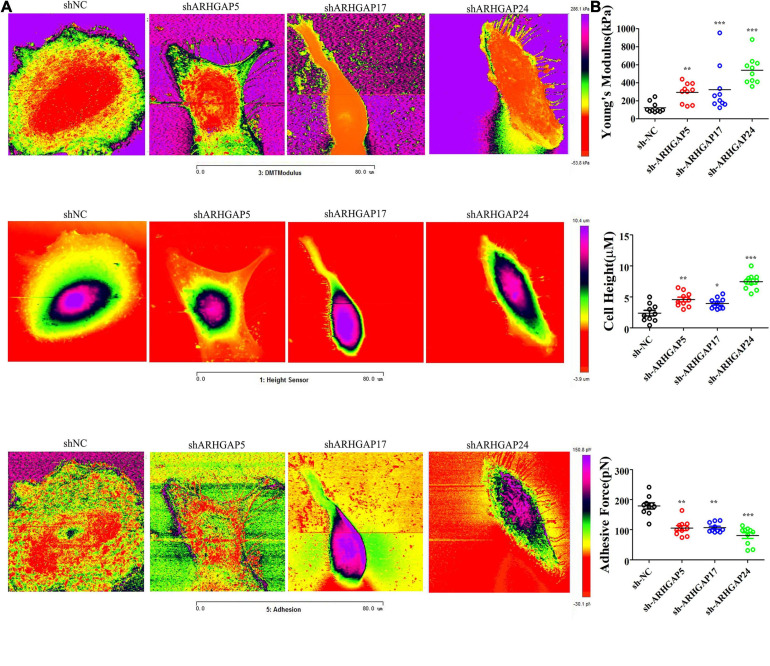
Identification the nanomechanical parameters *via* knocking down of ARHGAP5, ARHGAP17, and ARHGAP24 in BCa. **(A)** High-magnification images of the distinctive contrast patterns observed for T24 and knocking down ARHGAP5, ARHGAP17, and ARHGAP24 T24 cells. First-row images correspond to Young’s modulus (YM) maps. Second-row images correspond to cell height. Third-row images correspond to AF maps. **(B)** All cellular nanomechanical properties were analyzed in GraphPad Prism5 (*p < 0.05; **p < 0.01; ***p < 0.001; ns, not statistically significant, Wilcoxon signed rank test).

Cell motility, mostly determined by cytoskeleton mediated by ARHGAP-activated Rho GTPase, played a vital role in tumor invasion and metastasis as well as immune cell chemotaxis and infiltration. To further validate ARHGAP-related cell motility, we measured cellular mechanical properties of BCa (Hecht et al., 2012). Generally, cancer cells are typically less “stiff” than normal cells with a trend in a reduced Young’s modulus (YM) value and an increase in adhesive force (AF) *via* AFM quantifying cellular nanomechanical properties (Plodinec et al., 2012; Chen et al., 2013). Cells with a low YM are more likely to undergo transendothelial migration or translocation into the tissues under bloodstream pressure (Dufrêne and Pelling, 2013; Li et al., 2014). A higher AF contributes to cell adhesion in the microenvironment of targeted tissues (Park and Lee, 2014). Both topography and mechanical properties of T24 and T24-shARHGAP5, ARHGAP17, and ARHGAP24 were measured. T24 cells showed an isotropic well-spread shape, while sh-ARHGAP cells showed a more spindle-like or irregular shape with relative ARHGAP knocking down. As shown in the elastic maps, the cell edge of each cell showed a larger Young’s modulus value than the cell center, which corresponded to the area of cell nucleus. This high Young’s modulus of the cell edge is due to the substrate and is consistent with a previous report (Park and Lee, 2014; Weder et al., 2014; Figure 9A). We also measured irregular phalloidin, which indicates dysfunction of cytoskeleton system and reduction in cell motility, in ARHGAP5 and ARHGAP24 knocked down T24 cells. The T24 cells possess the lowest Young’s modulus, the highest adhesive force and cell height, while the T24-shARHGAP cells possess relatively higher Young’s modulus and lower adhesive force and cell height, suggesting that ARHGAP maintained malignancy-related cellular mechanical properties of BCa (Figure 9B).

## Discussion

Rho-GTPases are GTP-binding cytoskeletal proteins that were thought to construct mechanical support to the cell membrane for preserving biological processes (Kumar and Epstein, 2011). Regulation of Rho-GTPases can be highly related to cancer progression (Li et al., 2020). Rho-GTPase-activating proteins (ARHGAP) serve as negative regulators of Rho-GTPases, and alteration in the genes and transcriptomes of the ARHGAP family can exert a carcinogenesis effect through Rho-like GTPase dysregulation in several cancer types. ARHGAP7 downregulation correlates with unfavorable prognosis in breast cancer patients especially in metastatic lesions (Chen et al., 2019). In contrast, ARHGAP18 upregulation correlates with favorable prognosis in breast cancer (Humphries et al., 2017). ARHGAP15, induced *via* androgen, plays a tumor suppressor role (Takagi et al., 2018). ARHGAP regulates several pathways of cytoskeleton dynamic remodeling and assembling, which may exert an important role in immune cell migration, thus, eventually influencing tumor immune activity and infiltration. However, the role of the ARHGAP family has not been clearly identified in BCa, especially in immune-related BCa microenvironment, which has not been elucidated.

Through integrative analysis of ARHGAP genes in BCa *via* cBioPortal, Oncomine, and Gepia, we found that the ARHGAP family mutates at 10% level in BCa, and a relatively lower ARHGAP gene expression was observed in BCa. ARHGAP5, ARHGAP8, ARHGAP11A, ARHGAP17, ARHGAP24, ARHGAP37 (STARD13), and ARHGAP38 (STARD8) were identified as prognosis-related ARHGAP genes in BCa.

GeneMANIA, STRING, and MCODE analysis of neighboring and coexpression genes of the ARHGAP family followed by GO and KEGG functional enrichment identified axon guidance, focal adhesion, and leukocyte transendothelial migration to be associated with the ARHGAP family genes, and focal adhesion, chemokine signaling pathway, and T-cell receptor signaling pathways were enriched in ARHGAP coexpression genes. Surprisingly, the ARHGAP family genes and coexpression genes were found to influence several non-cytoskeleton-related pathways including the chemokine signaling pathway and T-cell receptor signaling pathway in the tumor immune microenvironment.

Immune cell infiltration in a cancer-associated immune microenvironment is closely related with prognosis and immunotherapy efficiency in several cancer types. High TILs often relate to a better prognosis than low TILs. Tumor-infiltrating CD4 + T cells, CD8 + T cells, tumor-associated macrophages (TAMs), and neutrophils were associated with patient prognosis and tumor chemosensitivity. We analyzed the correlation between prognosis-related ARHGAP family gene expression and the degree of immune cell infiltration in BCa using xCell and TIMER. Higher ARHGAP5 correlates with lower Th1/Th2 cell ratio, higher Treg cell, and lower M1 macrophage infiltration, which indicates a relative tumor-promoting microenvironment. Lower Th1/Th2 cell ratio, higher DC cell infiltration, and higher Treg cell infiltration were observed in ARHGAP17, ARHGAP24, and ARHGAP37 (STARD13), indicating a tumor-promoting microenvironment. Higher ARHGAP17, ARHGAP24, ARHGAP37 (STARD13), and ARHGAP38 (STARD8) expression positively correlated with T-cell exhaustion markers: PD-1, CTLA4, TIM3, GZMB, and LAG3, while ARHGAP5 and ARHGAP8 shows negative or absence correlation with T-cell exhaustion markers. The CD8 + T cell serves as the most crucial member of the immune-related tumor microenvironment. They mediate tumor cell-specific immune responses through themselves or interaction with other immune cells. High infiltration and low T-cell exhaustion marker of CD8 + T cells predict a higher response to chemotherapy and an overall better prognosis. We also observed a higher DC cell infiltration and a higher Treg cell infiltration in tumor-suppressing ARHGAP microenvironment, which indicates that the ARHGAP family gene is closely linked with tumor DC penetration. DCs increase tumor cell metastasis *via* enhancing Treg cells and suppressing CD8 + T-cell antitumor cytotoxicity. Collectively, we demonstrated that immune cell infiltration in prognosis-related ARHGAP family genes correlates with a tumor-promoting microenvironment.

**FIGURE 10:**
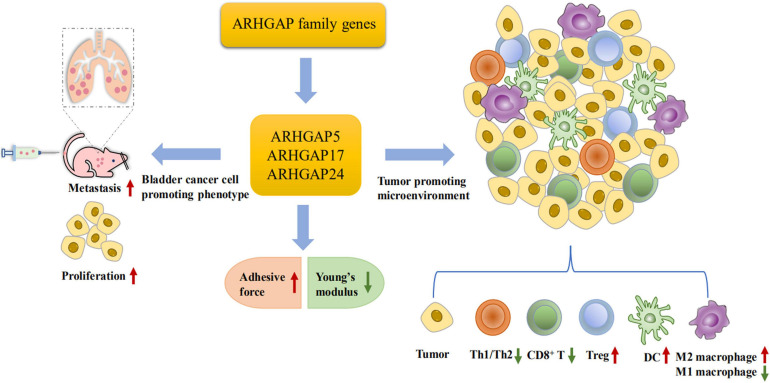
Scheme of ARHGAP family gene promotes BC progressing through establishing a tumor-promoting microenvironment and promotes BC cell proliferation, migration, and metastasis though cellular mechanical property-mediated cell motility.

Furthermore, we validated that silencing ARHGAP5, ARHGAP17, and ARHGAP24 suppressed BC cell proliferation, migration, and metastasis. Immune-related TGF-β, TNF-α, IL-2/STAT5, IL-6/JAK/STAT3, and the inflammatory response pathway and other signal pathways were related in ARHGAP5-, ARHGAP17-, and ARHGAP24-related BC carcinogenesis. Finally, since the different nanomechanical properties between normal and cancerous living cells enable cellular mechanical properties to be efficient markers for grading malignant-level cancer cells, we validated that T24-shARHGAP cells possess a relatively higher Young’s modulus and lower adhesive force and cell height, suggesting that ARHGAP maintains malignancy-related cellular mechanical properties of BCa. We still need to test the nanomechanical properties of immune cell mediated *via* knocking down ARHGAP. Taken together, we found that the ARHGAP family genes promote BC progressing through establishing a tumor-promoting microenvironment and promote BC cell proliferation, migration, and metastasis through cellular mechanical property-mediated cell motility (Figure 10).

## Data Availability Statement

The original contributions presented in the study are included in the article/Supplementary Material, further inquiries can be directed to the corresponding author/s.

## Ethics Statement

The animal study was reviewed and approved by Animal Care Committee of Fudan University.

## Author Contributions

HJ, YC, NL, and ZM were responsible for the conception and design, and study supervision. SW, QZ, and ZZ were responsible for the development of the methodology, analysis, and experiments. XC, YO, CY, CX, and XD performed the statistical and bioinformatic analysis. All authors read and approved the final manuscript.

## Conflict of Interest

The authors declare that the research was conducted in the absence of any commercial or financial relationships that could be construed as a potential conflict of interest.

## Footnotes

1 http://www.cbioportal.org

2 https://www.oncomine.org/resource/login.html

3 https://cistrome.shinyapps.io/timer/

4 http://gepia.cancer-pku.cn/

5 https://www.gsea-msigdb.org/gsea/msigdb

6 https://genemania.org/

7 https://string-db.org/

8 https://metascape.org/

9 https://cistrome.shinyapps.io/timer/

10 http://xCell.ucsf.edu/

## Supplementary Material

Supplementary Figure 1 Identification of expression profile and prognosis related ARHGAP family gene in BCa. **(A)** Expression level of ARHGAP family gene in several tumor and normal tissues in Oncomine. **(B)** Expression level of ARHGAP8, ARHGAP11A, ARHGAP37, and ARHGAP38 in several tumor and normal tissues and Expression, OS and DFS of ARHGAP8, ARHGAP11A, ARHGAP37, and ARHGAP38 in BCa.

Supplementary Figure 2 Functional enrichment analysis of ARHGAP family genes in BCa. **(A)** Correlation analysis of ARHGAP family genes. **(B,C)** Protein-protein interaction network of ARHGAP family genes and relevant co-expression genes through GeneMANIA and STRING analysis.

Supplementary Figure 3 Correlations between prognosis related ARHGAP family gene associated immune cell infiltration. **(A)** Immune infiltration correlation with ARHGAP8, ARHGAP11A, ARHGAP37, and ARHGAP38 in TCGA-BCa samples were carried out by TIMER. **(B)** Boxplots were used to visualize ARHGAP family associated certain cell type enrichment scores and logarithm transformed immune scores, stroma scores, microenvironment scores of different groups through xCell analyzed TCGA samples in BCa. **(C)** ARHGAP family associated infiltration of CD8+ T cells, CD4+ T cells, type 1 T helper (Th1) cells, type 2 T helper (Th2) cells, natural killer (NK) cells, Treg cells, M1 macrophages, M2 macrophages and Dendric cell were analyzed through xCell analyzed TCGA samples in BCa. Grouping was done according to the expression level of prognosis related ARHGAP family gene. (^∗^*p* < 0.05; ^∗∗^*p* < 0.01; ^∗∗∗^*p* < 0.001; ns, not statistically significant, Wilcoxon signed rank test).

Supplementary Figure 4 Gating strategy for CD8+ T cells.

## Abbreviations

AFM: atomic force microscopy
ARHGAP: Rho GTPase-activating protein
BCa: bladder cancer
BP: biological process
CC: cellular component
CCK8: Cell Counting Kit-8
DC: dendritic cell
DMEM: Dulbecco’s modified Eagle’s medium
EdU: 5-ethynyl-2′-deoxyuridine
EMT: epithelial–mesenchymal transition
FDR: false discovery rate
FBS: fetal bovine serum
FPKM: fragments per kilobase million
KEGG: Kyoto Encyclopedia of Genes and Genomes
GO: Gene Ontology
GTEx: Genotype-Tissue Expression
GSEA: gene set enrichment analysis
IL-2: interleukin 2
IL-6: interleukin 6
MCODE: Molecular Complex Detection
MF: molecular function
NES: normalized enrichment score
NK: natural killer
PFA: paraformaldehyde
PPI: protein–protein interaction
RhoA: Ras homolog family member A
TAMs: tumor-associated macrophages
TCGA: The Cancer Genome Atlas
TGF-β: transforming growth factor beta
TNF-α: tumor necrosis factor alpha
Th1: type 1 T helper
Th1: type 2 T helper
WB: Western blotting
YM: Young’s modulus.
