# Supplementary figures and images for: Transcriptomic Analysis Identified ARHGAP Family as a Novel Biomarker Associated With Tumor-Promoting Immune Infiltration and Nanomechanical Characteristics in Bladder Cancer
Source: Front Cell Dev Biol. 2021 Jul 7;9:657219. doi: 10.3389/fcell.2021.657219 (PMC8294098; doi:10.3389/fcell.2021.657219)

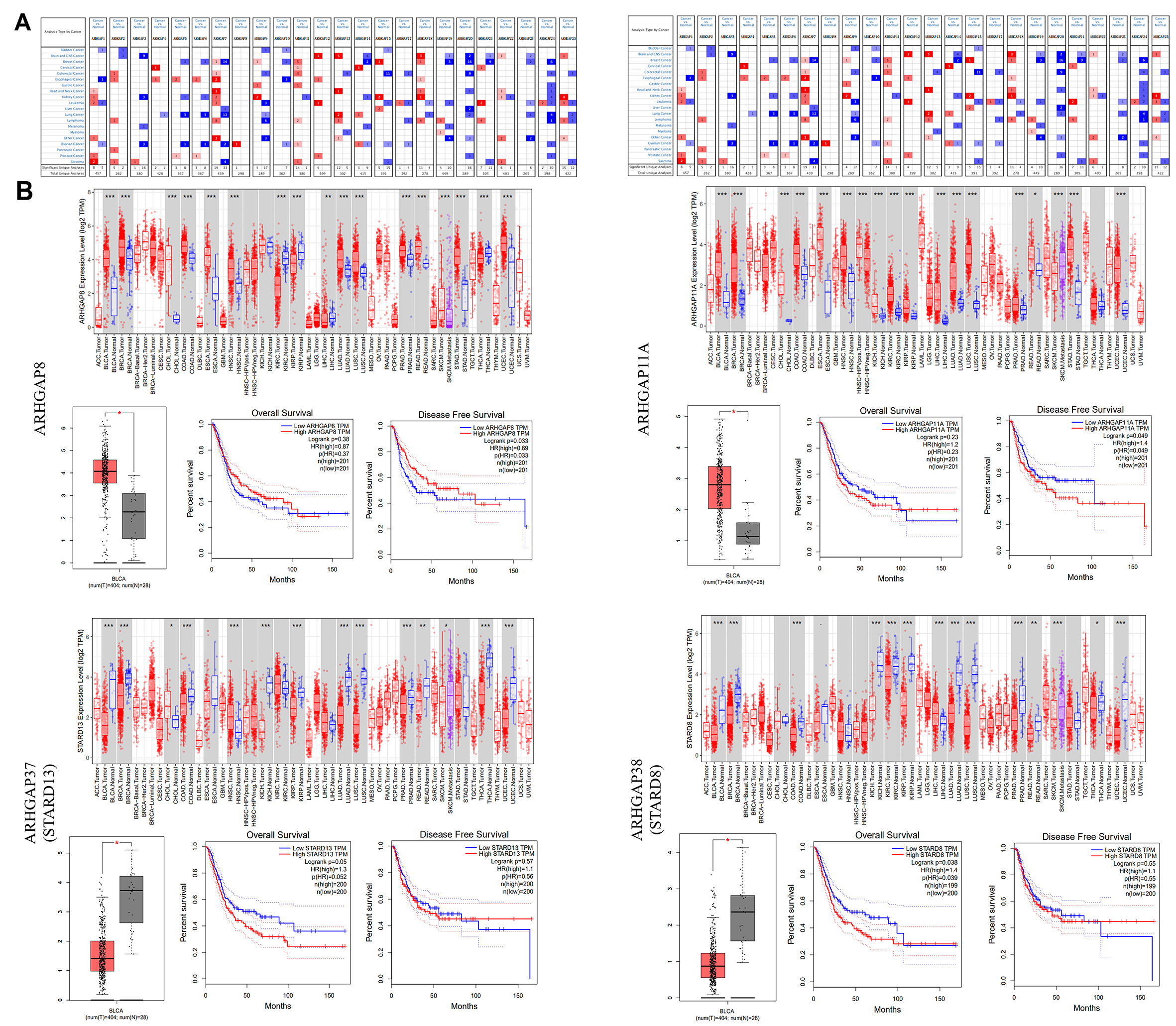

Supplement: Supplementary Figure 1 — Identification of expression profile and prognosis related ARHGAP family gene in BCa. (A) Expression level of ARHGAP family gene in several tumor and normal tissues in Oncomine. (B) Expression level of ARHGAP8, ARHGAP11A, ARHGAP37, and ARHGAP38 in several tumor and normal tissues and Expression, OS and DFS of ARHGAP8, ARHGAP11A, ARHGAP37, and ARHGAP38 in BCa. [file Image_1.TIF]

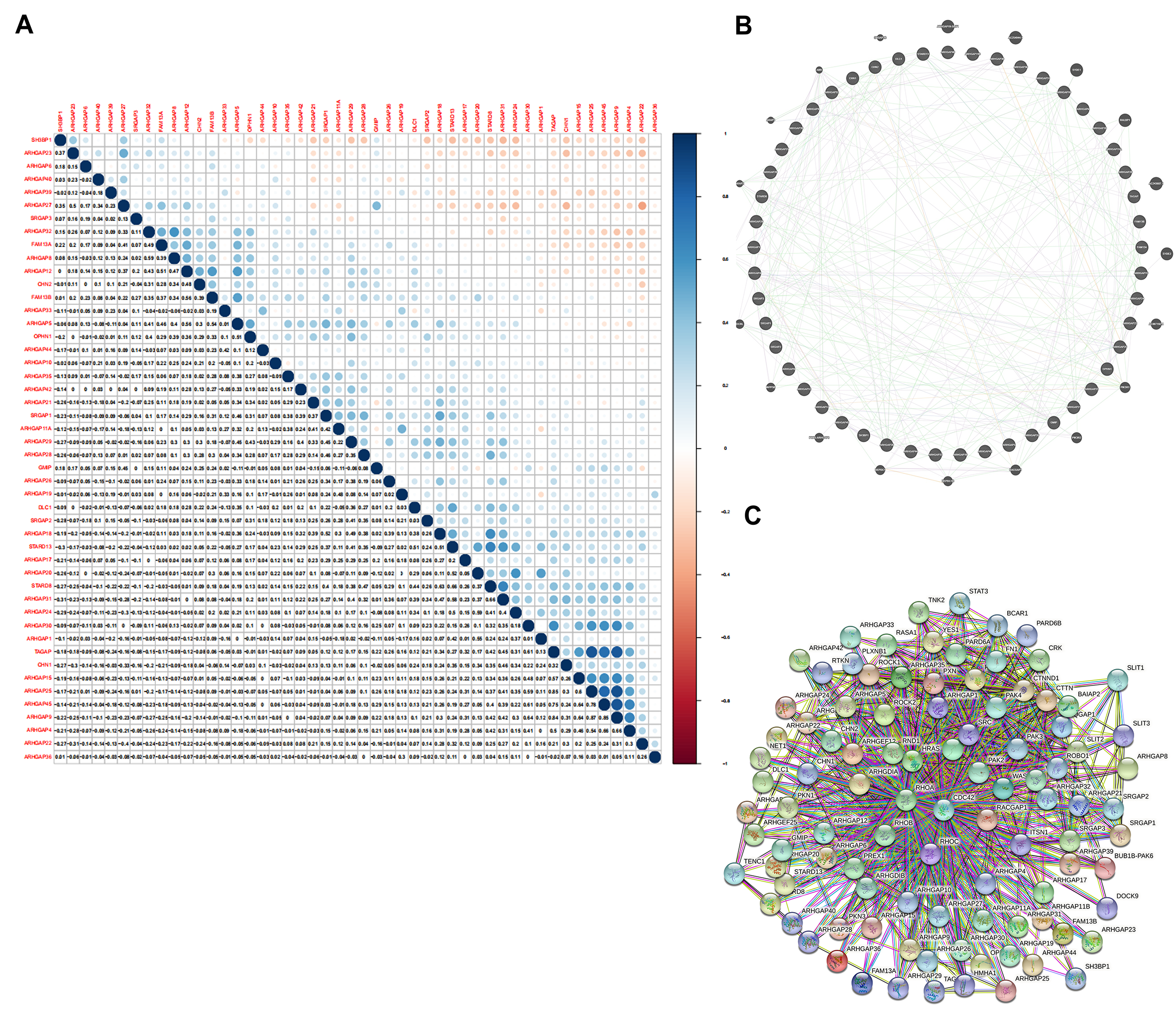

Supplement: Supplementary Figure 2 — Functional enrichment analysis of ARHGAP family genes in BCa. (A) Correlation analysis of ARHGAP family genes. (B,C) Protein-protein interaction network of ARHGAP family genes and relevant co-expression genes through GeneMANIA and STRING analysis. [file Image_2.TIF]

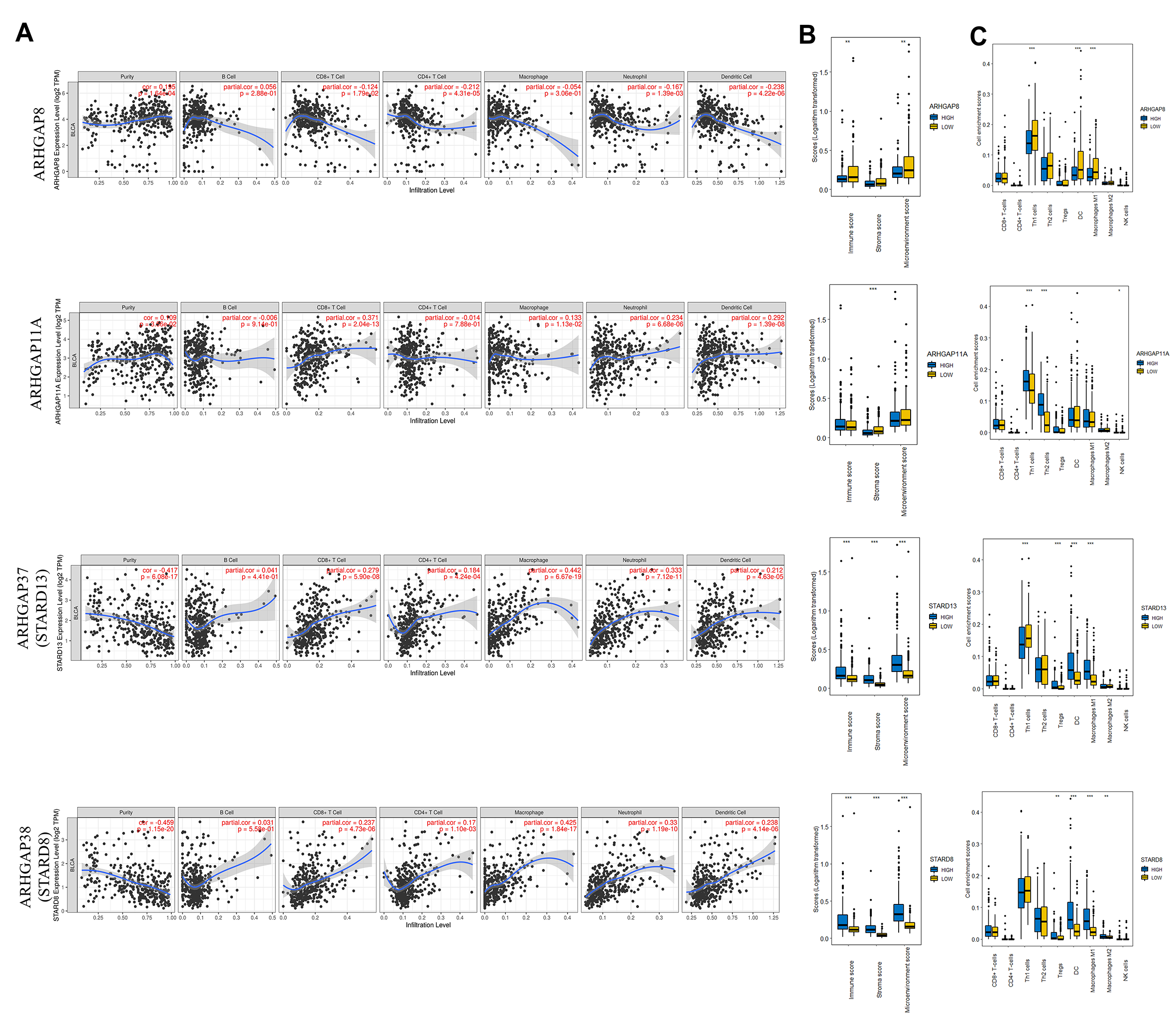

Supplement: Supplementary Figure 3 — Correlations between prognosis related ARHGAP family gene associated immune cell infiltration. (A) Immune infiltration correlation with ARHGAP8, ARHGAP11A, ARHGAP37, and ARHGAP38 in TCGA-BCa samples were carried out by TIMER. (B) Boxplots were used to visualize ARHGAP family associated certain cell type enrichment scores and logarithm transformed immune scores, stroma scores, microenvironment scores of different groups through xCell analyzed TCGA samples in BCa. (C) ARHGAP family associated infiltration of CD8+ T cells, CD4+ T cells, type 1 T helper (Th1) cells, type 2 T helper (Th2) cells, natural killer (NK) cells, Treg cells, M1 macrophages, M2 macrophages and Dendric cell were analyzed through xCell analyzed TCGA samples in BCa. Grouping was done according to the expression level of prognosis related ARHGAP family gene. (∗p < 0.05; ∗∗p < 0.01; ∗∗∗p < 0.001; ns, not statistically significant, Wilcoxon signed rank test). [file Image_3.TIF]

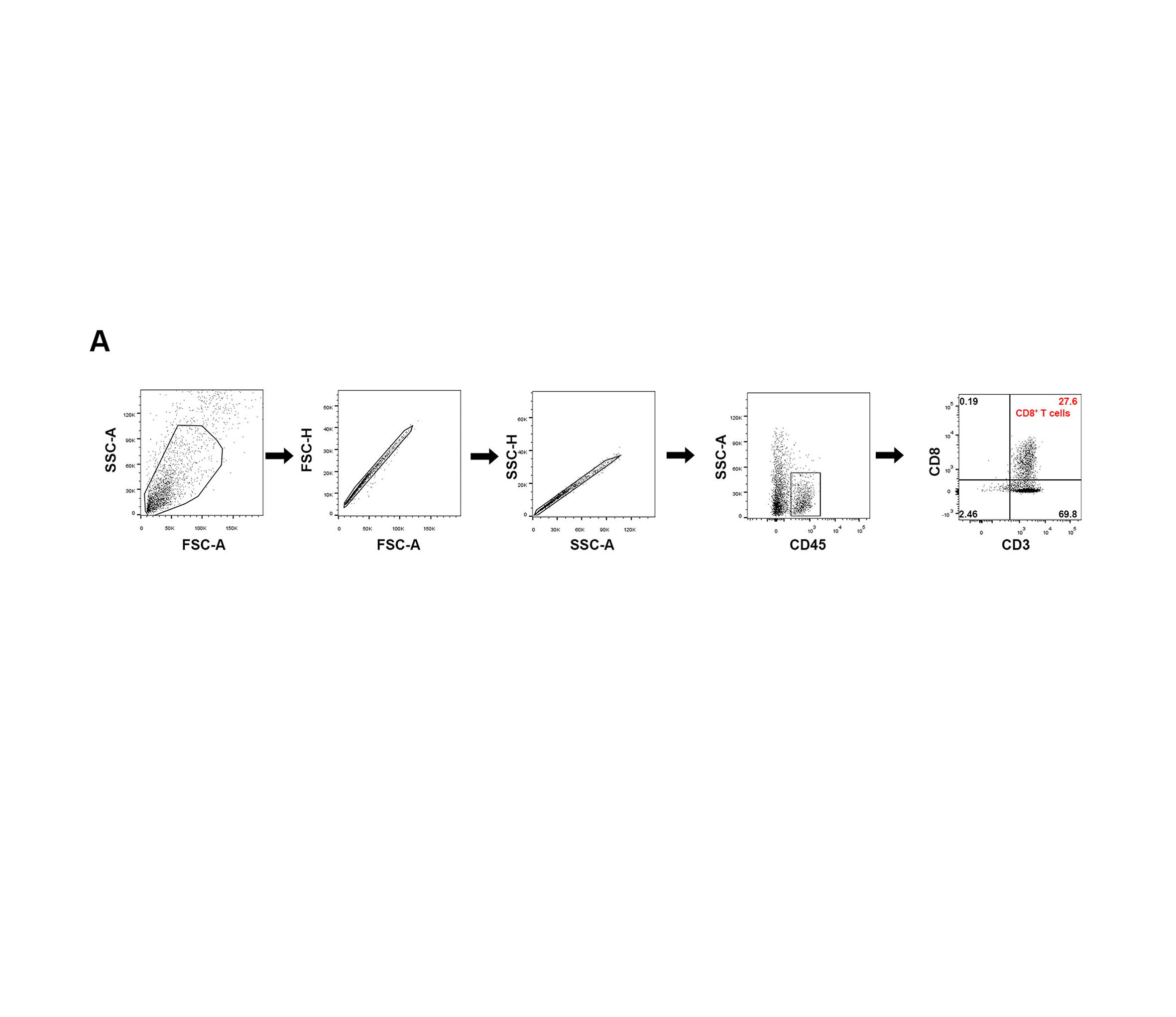

Supplement: Supplementary Figure 4 — Gating strategy for CD8+ T cells. [file Image_4.TIF]
