# Supplementary material for: Transcriptomic Analysis Identified ARHGAP Family as a Novel Biomarker Associated With Tumor-Promoting Immune Infiltration and Nanomechanical Characteristics in Bladder Cancer
Source: Front Cell Dev Biol. 2021 Jul 7;9:657219. doi: 10.3389/fcell.2021.657219 (PMC8294098; doi:10.3389/fcell.2021.657219)
Supplement: Supplementary file 5 [file Table_1.DOC]

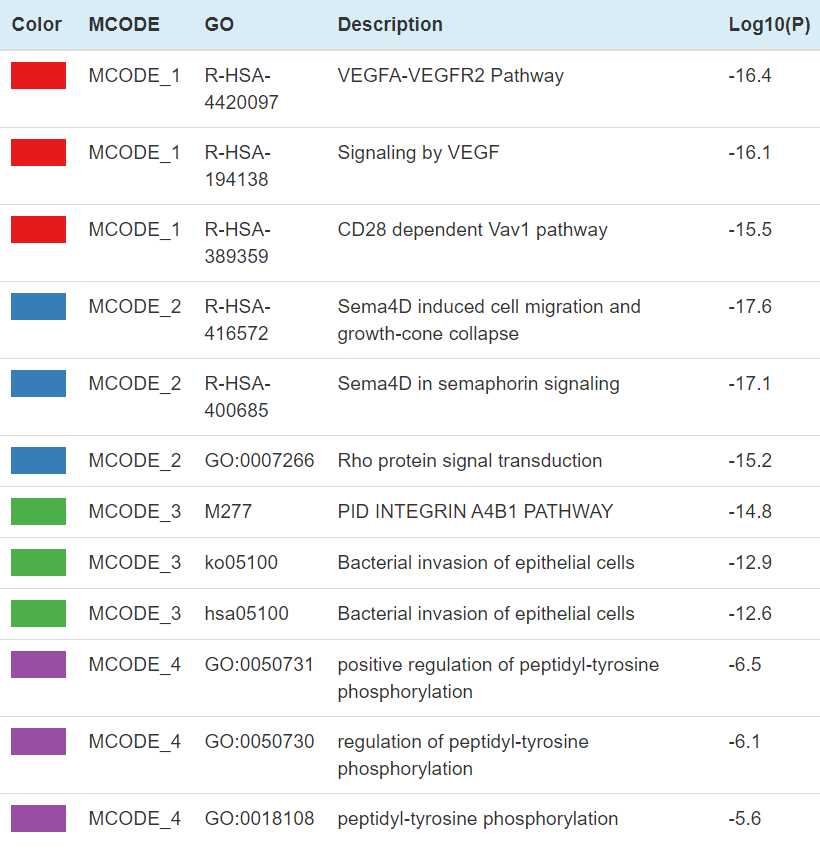


**Supplementary table 1**. Pathways and functional enrichment analysis were performed of each MCODE component to identify hub genes among the correlating genes independently. Each three most significant pathways of mcode were showed.
