# Supplementary material for: Transcriptomic Analysis Identified ARHGAP Family as a Novel Biomarker Associated With Tumor-Promoting Immune Infiltration and Nanomechanical Characteristics in Bladder Cancer
Source: Front Cell Dev Biol. 2021 Jul 7;9:657219. doi: 10.3389/fcell.2021.657219 (PMC8294098; doi:10.3389/fcell.2021.657219)
Supplement: Supplementary file 8 [file Table_4.DOC]

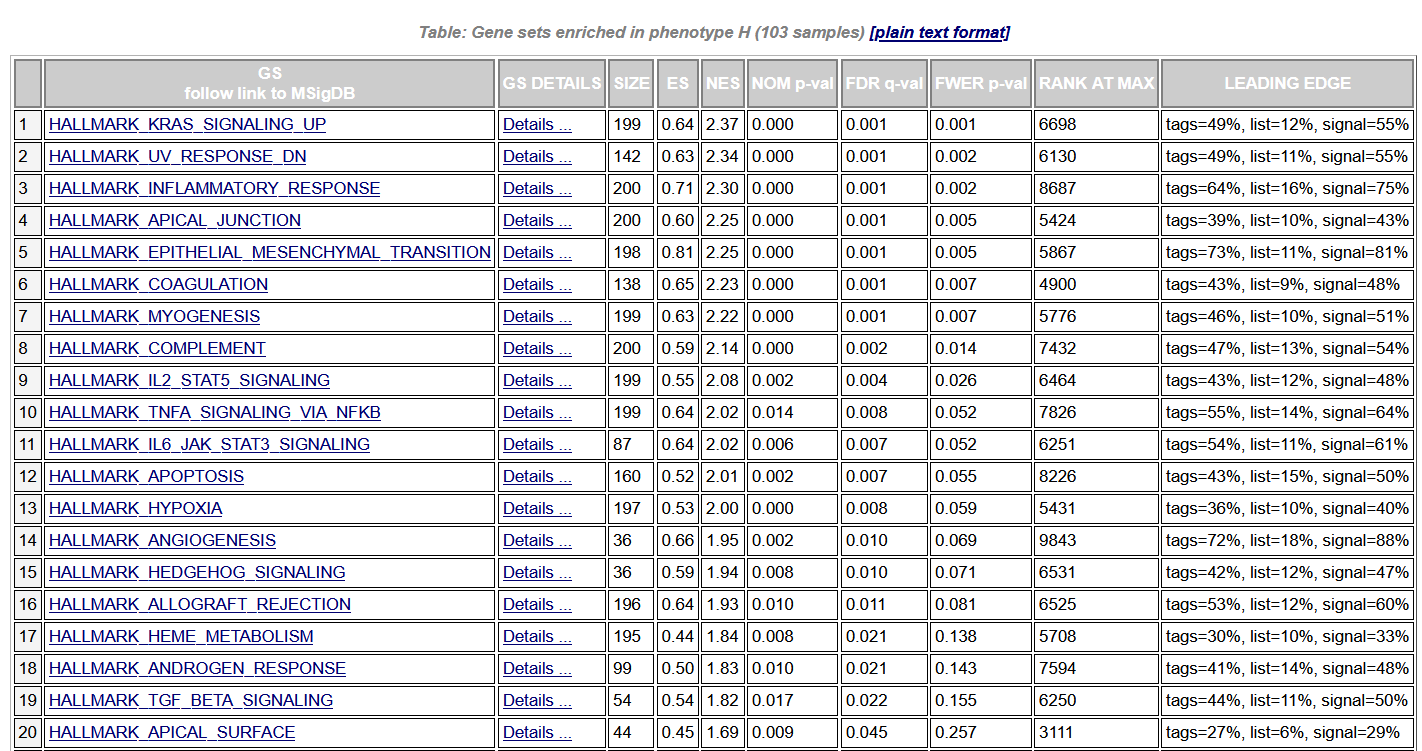


**Supplementary table 4**.Molecular mechanism of representative ARHGAP24 in BC biological process through GSEA enrichment plot of ARHGAP24 in TCGA cohort with p<0.05.FDR<0.25.
